# Fabrication of nanoparticles with precisely controllable plasmonic properties as tools for biomedical applications†

**DOI:** 10.1039/d4nr02677b

**Published:** 2025-01-15

**Authors:** Pauline Kolar-Hofer, Giulia Zampini, Christian Georg Derntl, Enrica Soprano, Ester Polo, Pablo del Pino, Nurgul Kereyeva, Moritz Eggeling, Leoni Breth, Michael J. Haslinger, Michael Mühlberger, Peter Ertl, Astrit Shoshi, Julian Hartbaum, Michael Jurisch, Beatriz Pelaz, Stefan Schrittwieser

**Affiliations:** a AIT Austrian Institute of Technology, Molecular Diagnostics 1210 Vienna Austria stefan.schrittwieser@ait.ac.at; b Centro Singular de Investigación en Química Biolóxica e Materiais Moleculares (CiQUS), Universidade de Santiago de Compostela 15782 Santiago de Compostela Spain; c Centro Singular de Investigación en Química Biolóxica e Materiais Moleculares (CiQUS), Departamento de Bioquímica, Universidade de Santiago de Compostela 15782 Santiago de Compostela Spain; d Centro Singular de Investigación en Química Biolóxica e Materiais Moleculares (CiQUS), Departamento de Física de Partículas, Universidade de Santiago de Compostela 15782 Santiago de Compostela Spain; e Department for Integrated Sensor Systems, University for Continuing Education Krems 2700 Wr. Neustadt Austria; f PROFACTOR GmbH Im Stadtgut D1 4407 Steyr-Gleink Austria; g Institute of Applied Synthetic Chemistry, Institute of Chemical Technologies and Analytics, Technische Universitaet Wien (TUW) Vienna Austria; h Institut für Mikroelektronik Stuttgart (IMS CHIPS) Allmandring 30a 70569 Stuttgart Germany; i Centro Singular de Investigación en Química Biolóxica e Materiais Moleculares (CiQUS), Departamento de Química Inorgánica, Universidade de Santiago de Compostela 15782 Santiago de Compostela Spain beatriz.pelaz@usc.es

## Abstract

Metal nanoparticles are established tools for biomedical applications due to their unique optical properties, primarily attributed to localized surface plasmon resonances. They show distinct optical characteristics, such as high extinction cross-sections and resonances at specific wavelengths, which are tunable across the wavelength spectrum by modifying the nanoparticle geometry. These attributes make metal nanoparticles highly valuable for sensing and imaging in biology and medicine. However, their widespread adoption is hindered due to challenges in consistent and accurate nanoparticle fabrication and functionality as well as due to nanotoxicological concerns, including cell damage, DNA damage, and unregulated cell signaling. In this study, we present a fabrication approach using nanoimprint lithography in combination with thin film deposition which yields highly homogenous nanoparticles in size, shape and optical properties with standard deviations of the main geometry parameters of less than 5% batch-to-batch variation. The measured optical properties closely match performed simulations, indicating that pre-experimental modelling can effectively guide the design of nanoparticles with tailored optical properties. Our approach also enables nanoparticle transfer to solution. Particularly, we show that the surface coating with a PEG polymer shell ensures stable dispersions in buffer solutions and complex cell media for at least 7 days. Furthermore, our *in vitro* experiments demonstrate that these nanoparticles are internalized by cells *via* endocytosis, exhibit good biocompatibility, and show minor cytotoxicity, as evidenced by high cell viability. In the future, our high-precision nanoparticle fabrication method together with tunable surface plasmon resonance and reduced nanotoxicity will offer the possibility to replace conventional nanomaterials for biomedical applications that make use of an optical response at precise wavelengths. This includes the use of the nanoparticles as contrast agents for imaging, as probes for targeted photothermal cancer therapy, as carriers for controlled drug delivery, or as probes for sensing applications based on optical detection principles.

## Abstract 2

Highly homogenous nanoparticles fabricated *via* nanoimprint lithography with minimal batch-to-batch size variation and tailor-made plasmonic properties. Polymer surface modification ensures functionality, good biocompatibility and minor cytotoxicity.

## Graphical abstract

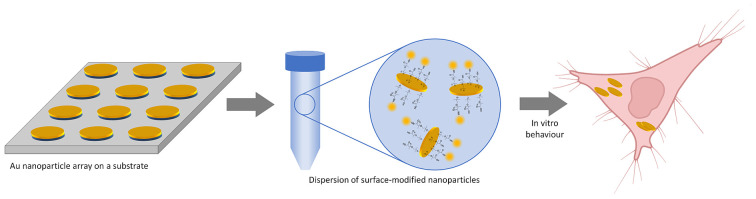

## Introduction

A prominent, and still growing, field of applications of engineered nanoparticles (NPs) is their utilization for medical and biological use.^1,2^ In particular, metal-based nanomaterials represent a type of NPs that are already well established, especially for biomedical applications.^3^ Within this field, NPs with distinct optical properties are of special interest.^4^ Here, localized surface plasmon resonances are one of the main physical phenomena that are responsible for specific optical properties such as distinct resonance wavelengths or a high extinction and absorption cross-section.^5^ Additionally, the optical resonances are tuneable across the wavelength spectrum of light by alterations of the NP geometry. This allows to achieve high extinction cross sections in the near-infrared region of light where biological tissue is highly transmissive.^5^ Due to their unique properties, these NPs are of high interest for sensing and imaging in biology and medicine.^6–11^ However, one main factor hindering metal NPs to be regularly employed as standard tool for biomedical applications lies in their nanotoxicology behaviour, especially as many types of NPs are known to be involved in cell damage, DNA damage, or unregulated cell signalling.^12–14^ Decisive for their cytotoxic behaviour are the size, the structure, the shape and surface properties of the NPs of interest.^12^

In literature, nanoparticles are typically considered as highly homogeneous if the standard deviation of the measured size is less than 10%, which is hard to accomplish – even for optimized synthesis routes.^15–17^ Nanoparticle dispersions with particle shapes that differ from simple spheres show a significant amount of residual spherical particles in the final nanoparticle solution, which is due to the common synthesis approaches starting from spherical seed particles to grow alternative shapes. The most sophisticated approaches of synthesizing monodisperse elongated nanoparticles have an amount of 3% of the particles remaining in a spherical shape.^18^ Furthermore, Kumar *et al.* state in their recent review that coherent NP fabrication and functionality for specific applications is currently the real scientific challenge.^10^ These points are the focus in the current manuscript. Numerous methods are reported in the literature for the fabrication of metal NPs and cover both, top-down and bottom-up fabrication techniques.^19^ Nanoimprint lithography (NIL) is a top-down nanofabrication technique to replicate nanostructures on a surface.^20–22^ One of the primary advantages of NIL is its ability to produce highly uniform and reproducible nanostructures. This precision is crucial for biological applications, where consistency and accuracy are essential for reliable results, which is fundamental to understand their biological behaviour. NIL can be applied together with thin film deposition methods to fabricate NPs.^23–27^ In a first step of the NP fabrication, a polymer layer is applied onto the surface of a substrate material. In a next step, a nanostructured stamp is pressed into the polymer layer. After curing of the polymer, the stamp is removed, and a negative image of the nanostructures is obtained in the polymer layer.^28–30^ Ultra-violet NIL (UV-NIL) uses a photocurable resist as a polymer layer and an ultra-violet light source to cure the resist.^30–33^ This way, nanostructures with feature sizes down to the low nanometre regime can be fabricated.^34^

**Fig. 1:**
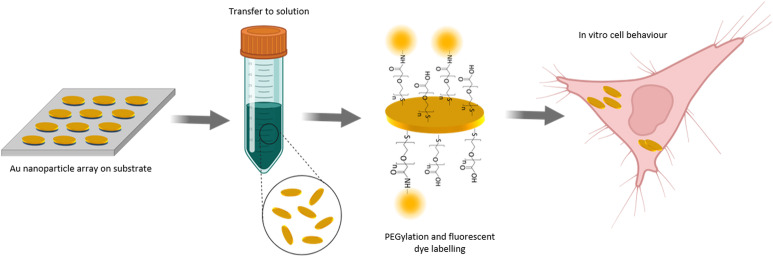
Conceptual illustration of the nanoparticle fabrication based on nanoimprint lithography and surface modification by wet-chemistry methods. Gold nanoparticles with the shape of elliptical platelets were fabricated on a flat substrate and then transferred to solution *via* a wet-chemical etching step of an underlying sacrificial layer. The nanoparticles were surface modified by coating with a heterobifunctional PEG polymer which allows for further surface modification by a fluorophore. The nanoparticles were internalized by living cells allowing to assess their *in vitro* behaviour.

In this paper, we present a NP fabrication approach which enables the production of NPs with precisely defined geometry and high homogeneity in terms of size, shape and optical properties (Fig. 1). Our NP fabrication yielded highly homogenous NPs with standard deviations of the main geometry parameters of less than 5% across various batches. Remarkably, as shown in the manuscript below, the experimentally examined optical properties match very well the simulated ones. This aspect is of high importance as it implicates that simulations prior to experiments can be applied to design the geometrical features of the stamp in order to obtain nanomaterials with tailor-made optical properties. We show that our fabrication approach allows NP transfer to solution. Surface coating by a PEG polymer shell results in the formation of NP dispersions that are stable in buffer solutions and complex cell media for at least 7 days. Additionally, we show that our NPs are internalized by cells *via* endocytosis and that good biocompatibility and minor cytotoxicity of the NPs was achieved as indicated by the high cell viability in *in vitro* experiments.

## Results and discussion

The NP fabrication method developed here is based on UV-NIL and thin film deposition methods and involves wet-chemical as well as dry-physical etching steps as detailed below. One critical and fundamental step to produce nanomaterials for biological applications is their surface coating. One of the most common and best-established methods is the coating of the NP surface with polyethylene glycol (PEG), also referred as PEGylation.^35,36^ PEGylation is known to improve biocompatibility and to have a positive effect on nanoparticle-cell interaction such as diminished cytotoxicity.^37,38^ In therapeutic and diagnostic applications, PEG is considered to be one of the most favourable polymers for NP surface modification as it is providing longer circulation times.^39^ In terms of bioconjugation, *i.e.* the linking of a biomolecule to the NP, PEG chains are available presenting different reactive groups. Also, heterobifunctional PEG chains can be used to apply bioconjugation reactions to provide the NPs with extra functionalities or biologically relevant molecules. Consequently, we have chosen to modify our NP surface with a PEG polymer shell.

The overall concept of our developed NP fabrication and surface modification route is shown in Fig. 1. In a first step, gold NPs with the shape of elliptical platelets were fabricated as an array on a substrate. The deposition of a sacrificial layer underneath the gold and its later dissolving by wet-chemical etching allowed NP transfer from the substrate to solution. Surface modification of the NPs was done by coating them with a heterobifunctional PEG polymer. This allowed for further surface modification by a fluorescent dye. The so fabricated NPs were incubated with cells to allow for cell uptake and to study the *in vitro* behaviour of the NPs with respect to nanotoxicity.

**Fig. 2:**
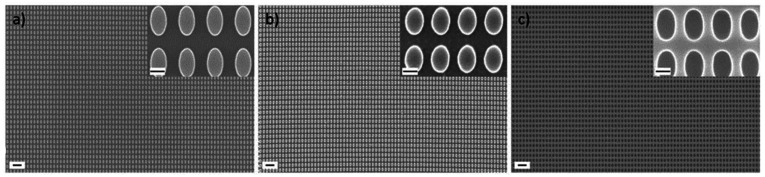
Scanning electron microscopy (SEM) images. Overview at lower magnification and detailed image at higher magnification (inset in the top right). (a) Si master stamp. (b) Polydimethylsiloxane (PDMS) stamp. (c) UV-NIL imprinted nanostructure. The scale bars correspond to 1 μm for the large overview images and to 200 nm for the insets showing details at higher magnification.

In a first step, a Si master stamp was fabricated using electron beam lithography and various etching processes as detailed in the Methods section. Specifically, a nanostructured array of elliptical pillars with ellipse dimensions of 400 nm in length and 200 nm in width was patterned. Scanning electron microscopy (SEM) images of the master stamp are shown in Fig. 2a and Fig. S1.† This master stamp was replicated in a UV curable resist to form a first intermediate imprint (see Fig. S2† for SEM images of the intermediate imprint). In the next step, we fabricated a polydimethylsiloxane (PDMS) stamp by replicating the intermediate imprint. PDMS stamps are routinely applied in UV-NIL as they enable the replication of small features sizes down to 10 nm.^40^ Furthermore, PDMS stamps are transparent for UV light, possess good gas permeability to absorb trapped air during the imprint process and also provide some mechanical flexibility when being pressed into the resist, which facilitates conformal contact as well as easy release of the stamp.^41^ SEM images of the PDMS stamp, see Fig. 2b, confirm that the well-ordered array of elliptical pillars is preserved. With this stamp, we performed multiple replications using UV-NIL on a NIL resist coated silicon wafer substrate. Due to the additional initial intermediate imprinting step, the resulting nanostructure is a negative copy of the original Si master stamp and, thus, shows a resist layer with elliptical holes (see Fig. 2c for SEM images). A photograph of a substrate with 28 individual imprints is shown in Fig. S3.†

As mentioned above, the NPs were fabricated inside the elliptical holes of the imprinted structure. To maximize the homogeneity of the NP geometry, we applied dry-physical and wet-chemical etching processes that created pockets in the nanostructured resist film (see Fig. S4†). This procedure ensured NP growth directly on the flat Si wafer substrate surface and avoided a physical connection of the NPs, especially their edges, to the resist film. Otherwise, this would result in uneven and jagged NP edges after resist lift-off. In the absence of a well-shaped pocket, the NP would also fully or partially grow on the resist film. As a result, the lift-off process would then break the bond between the resist and the NP, which would affect the homogeneity of the NP shape and geometry. Schematics and SEM images of the etching processes and the fabricated pockets are shown in Fig. S4,† and process details are given in the Methods section.

**Fig. 3:**
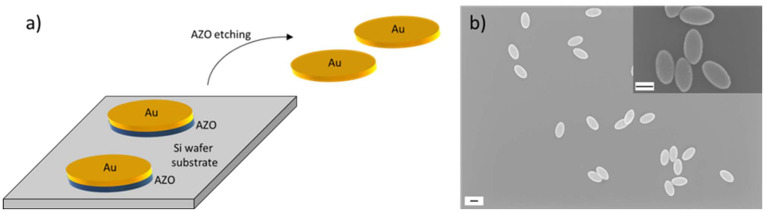
Sketch of the nanoparticle transfer to solution and SEM images of the nanoparticles. (a) Sketch of gold (Au) nanoparticle transfer from the silicon wafer substrate to solution *via* etching of the sacrificial aluminium-doped zinc oxide (AZO) layer. (b) SEM images of the nanoparticles after drop-casting from solution onto an imaging substrate. Overview at lower magnification and detailed image at higher magnification (inset in the top right). The scale bars correspond to 200 nm.

In a next step of the NP fabrication route, a gold layer was deposited onto the nanoimprinted substrate by thermal evaporation and analysed using SEM. We primarily focus on gold NPs with 30 nm thickness, but also examined NPs with thicknesses of 20, 40, 50, and 60 nm. Fig. S5† shows schematics and a SEM image of the nanostructure after formation of the NPs, which clearly demonstrates the above discussed formation of pockets in the resist film that contain the NPs. To allow the later release of the NPs from the substrate and to transfer them to solution, a sacrificial layer underneath the gold layer was deposited. As sacrificial layer, we have chosen aluminium-doped zinc oxide (AZO) which was sputter deposited onto the imprinted substrate before the gold evaporation took place. AZO was chosen because it can be easily etched and dissolved in basic conditions. Furthermore, the deposition of AZO resulted in a low surface roughness with a measured mean root square roughness of 224 pm, which is a pre-condition for smooth gold NPs as they are fabricated on top of the AZO layer. Fig. S6† shows scanning force microscopy (SFM) images of three AZO layer thicknesses employed to measure the respective surface roughness. We have chosen the AZO thickness to be 25 nm as a compromise between a smooth surface and a quick and reliable etching rate to release the NPs from the substrate. A schematic showing the gold NPs with the sacrificial layer underneath and the release from the substrate by wet-chemical etching of the AZO layer is shown in Fig. 3a. After this step, NP dispersions were obtained. A SEM image of the resulting gold NPs is presented in Fig. 3b and SEM-EDX analysis in Fig. S7.† We fabricated individual dispersions with NPs of various thicknesses, *i.e.* 20 nm, 30 nm, 40 nm, 50 nm, and 60 nm. We evaluated the lateral dimensions of the fabricated NPs by analysis of SEM images and determined a full long ellipse axis of 376 ± 12.2 nm and a full short ellipse axis of 204 ± 10 nm. This means that the NP fabrication yielded highly homogenous NPs with standard deviations of the main geometry parameters of less than 5%. The mean value and the standard deviation were calculated across all batches of fabricated NP thicknesses. The variations of NPs with a thickness of 30 nm which were employed for the investigation of cell interactions in the current work are shown in Fig. S8.† Though the NP geometry of 376 × 204 nm matched the dimensions of the structure on the Si master stamp of 398 × 205 nm very well, a slight deviation was obtained. The reason is twofold. First, the pillars of the master were not perfect straight columns, but conical in their shape. This is shown by the SEM images in Fig. S1.† SEM image analysis revealed ellipse dimensions at the top of the pillars of 398 × 205 nm and 440 × 240 nm at the pillar bottom. After conducting the two imprint steps (intermediate and UV-NIL imprint), ellipse dimensions of 425 × 253 nm were obtained. Therefore, the difference between the imprint ellipse geometry and the NP geometry amounts to about 40 nm in both ellipse axes. The second reason why the ellipse dimensions on the Si master stamp slightly differed from the dimensions of the fabricated NPs was in the nature of the sputter deposition process that was employed to fabricate the sacrificial AZO layer. The target material was placed in a distance to the sample so that, as a result, the deposition was isotropic and it led to the coating of an AZO layer on the side walls of the elliptical holes. This caused the elliptical holes in the imprint to decrease in lateral size after the sputter deposition so that the formation of the gold NP was only possible inside the reduced elliptical nanohole. The difference of the ellipse dimensions of 49 nm very well matches the thickness of the deposited AZO layer when considering the isotropic deposition on all side walls of the elliptical hole (25 nm on both ends of the ellipse axes).

For the characterization of the optical properties of the fabricated NP dispersions, we conducted simulations of the optical NP properties to obtain the extinction efficiency factor for each fabricated NP type using software based on the method of the discrete dipole approximation (DDA).^42^ The DDA simulation method is based on emulating the NP by an array of polarizable points, which interact with each other *via* the electric field of the incident excitation light. In a first step, we conducted a convergence study to determine a suitable dipole spacing (see Fig. S9†) and concluded to use a narrow dipole spacing of 2 nm. An example of a target serving as input for the simulation is shown in Fig. S10.† The simulations revealed the extinction efficiency factors for a random NP orientation in unpolarized light, which ensures comparability to measured absorbance values of a NP dispersion.

**Fig. 4:**
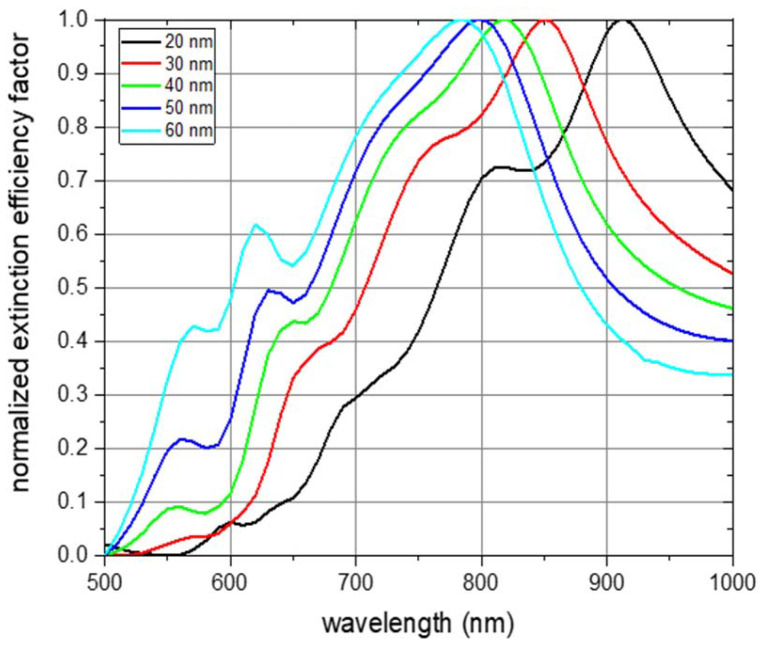
Simulation results of the optical extinction efficiency factor for varying nanoparticle thicknesses in dependence of the wavelength. Nanoparticle thickness of 20 nm is shown in black, 30 nm in red, 40 nm in green, 50 nm in blue, and 60 nm in cyan. Nanoparticles are oriented randomly, the excitation light is unpolarized, and the extinction efficiency factors are normalized.

Simulations of the extinction efficiency factor depending on the wavelength were run for each NP thickness. Normalized spectra for unpolarized incident light and randomly oriented NPs are shown in Fig. 4. The absorbance curve and the individual curve features are shifted towards smaller wavelengths with increasing NP thickness (black line for a NP thickness of 20 nm, red for 30 nm, green for 40 nm, blue for 50 nm, and cyan for 60 nm). The main absorbance peak in the visible and near-infrared region of light is shifted from about 900 nm for a particle thickness of 20 nm to about 780 nm for a thickness of 60 nm. This is an expected behaviour as the aspect ratios with respect to the three main NP axes are decreasing with increasing NP thickness.^43,44^ The origin of the main absorbance peak can be found in plasmon resonance modes along the short ellipse axis. This is depicted in Fig. S11,† which shows the absorbance spectra of the 6 main NP orientations for each of the examined NP thicknesses under excitation of linearly polarized light up to 1600 nm wavelength. The absorbance peak at about 550 nm which gets more pronounced with increasing thickness can be attributed to plasmon resonance modes along the thickness of the NP platelet. Spectra features between about 600 nm and 800 nm result from an overlay of features arising from excitations along the two main ellipse axes with dominant short ellipse axis contribution (see Fig. S11†). To exclude that these features arise from edge effects of the simulated NP geometry, we also conducted simulations of rounded ellipsoids of comparable geometry and observed a similar behaviour (see Fig. S12†). The main plasmon resonance peak along the long ellipse axis was found in the infrared region of light, which is shown in Fig. S11.† It varies between 1400 nm and 1550 nm, but, is not of further interest within the scope of the current work as biological applications are favoured in the visible and especially the near-infrared region of light due to the strong absorbance of water above about 1000 nm.^45^

**Fig. 5:**
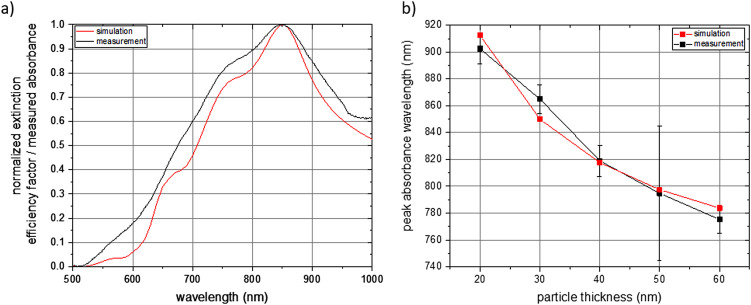
Comparison of the simulated (red line) and measured (black line) absorbance spectrum. (a) Normalized simulated and measured spectra for a nanoparticle with 30 nm thickness. (b) Peak absorbance wavelengths in dependence of the nanoparticle thickness. For both images, the nanoparticles are oriented randomly, and the excitation light is unpolarized for the simulation as well as for the measurement.

For the experimental validation of the simulated spectrum data, we did optical spectroscopy of the individual aqueous NP dispersions and determined the absorbance in dependence of the wavelength in the visible and near-infrared region of light. A comparison of a simulated and measured spectrum for NPs with a thickness of 30 nm is shown in Fig. 5a. Both spectra are normalized and reflect NPs with random orientation under excitation of unpolarized light. The measured values meet the simulated ones very well with respect to the wavelength of maximum absorbance and with respect to the individual features of the spectrum. In turn, this also means that *a priori* simulations can be employed to determine the geometry of a NP with tailor-made optical properties. Furthermore, it shows that the here presented NP fabrication based on UV-NIL can be applied, within the constraints of the fabrication method, for an experimental realization of a NP with a geometry determined by *a priori* simulations. A comparison of simulated and measured spectra regarding the other fabricated NP thicknesses is shown in Fig. S13.† It can be seen for all examined NP thicknesses that the simulations well match the experimental data. Deviations of the experimental values from the simulated ones are more pronounced for the NPs with the lowest thickness of 20 nm and for higher thicknesses of 50 nm and 60 nm. With respect to the 20 nm thick NPs, we attribute the difference between the simulated and the measured spectra to the fact that a small deviation of the deposited gold layer from the target of 20 nm represents a big relative difference in comparison to a larger thickness. This would result in different NP geometries for the simulation and the measurement and, thus, in differences in the optical spectra. Additionally, we expect a smoother surface of the gold for thicker NPs. Reason is that the NPs were grown on the sacrificial AZO layer and the surface roughness of the AZO layer translates to the roughness of the gold NP, which has a higher relative impact on thin layers than on thicker gold layers. Regarding the higher thicknesses of 50 nm and 60 nm, we attribute the deviations of the measured spectra from the simulated ones to deviations of the NP geometry from the perfect platelet, which are higher for increasing NP thickness. This is due to the imperfect anisotropic deposition of gold during the thermal evaporation step, which results in the gold coverage of the side walls of the elliptical holes. Coating of the side walls reduces the elliptical hole dimensions with increasing deposited gold thickness and results in NPs with a conical shape. This conical shape differs from the elliptical platelet employed as input parameter for the simulations leading to a deviation of the measured spectrum from the simulated one. A comparison of the simulated and measured wavelength of the absorbance maximum of all fabricated NPs with various thicknesses is shown in Fig. 5b. Again, the simulated data very well matches the measured spectra. As already discussed above for Fig. 4, the wavelength of the absorbance maximum shifts towards shorter wavelengths with increasing NP thickness.

For studying the NP dispersion stability and nanotoxicity, we have chosen the particles with a thickness of 30 nm. The aqueous stable dispersion of the particles was successfully achieved by surface derivatization with heterobifunctional polyethylene glycol (PEG) chains of 5 kDa. These PEG chains featured a thiol group (–SH) at one end to exploit gold–thiol binding mechanisms and a terminal carboxylic group (–COOH) at the other end so that the overall polymer is an HS-PEG-COOH molecule. The presence of terminal carboxylic groups allowed for electrostatic stabilization of the NP dispersion due to the deprotonation of the carboxylic group. Additionally, it also enabled further covalent immobilization of molecules such as fluorescent reporters. This covalent immobilization was essential for facilitating the monitoring of the behaviour of particles in living cells.

Our protocol aimed to ensure a homogeneous PEG coverage of the particles, retaining their optical properties and morphology while providing colloidal stability and reactive chemical groups on the particle surface.^37^ As mentioned above, the carboxylic groups enhanced colloidal stability in biological environments while serving as reactive sites for biding relevant molecules.^46^ The length of a 5 kDa PEG chain provided sufficient distance between the NPs’ surface and the labelled dyes, preventing luminescence quenching known to occur with fluorophores too close to the gold surface while ensuring colloidal stability in complex relevant biological media.^47^

The PEGylation protocol involved adding PEG in a significant excess to ensure complete surface coverage and replacement of potentially adsorbed residues on the particle surface resulting from the NP lift-off process transferring the particles from the substrate to solution. The maximum theoretical amount of PEG chains that could be adsorbed on the surface of the particles (PEG_(max)_/NP) was fixed at 1.5 PEG chains per theoretical nm^2^ of surface of each NP (see Table S1† for further details). This resulted in approximately 217 500 PEG chains per NP. For the PEGylation protocol, we decided to increase this value more than 20-fold, yielding a ratio of 5000 000 PEG chains per NP (PEG/NP).

**Fig. 6:**
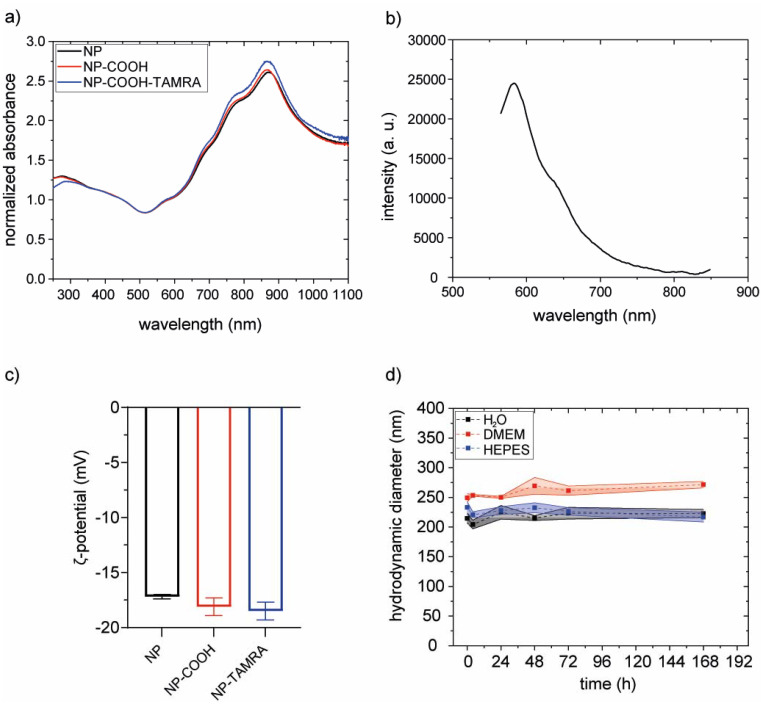
(a) Normalized (@450 nm) extinction spectra of bare NPs (black line), after PEGylation (NP-COOH, red line) and after TAMRA labelling (NP-COOH-TAMRA, blue line). (b) Emission spectrum of NP-COOH-TAMRA (*λ*_exc_ = 530 nm). (c) Graphical representation of zeta potential values of bare NPs (−17.2 ± 0.2 mV, black bar), NP-COOH (−18.1 ± 0.8 mV, red bar) and NP-COOH-TAMRA (−18.5 ± 0.8 mV, blue bar). (d) Colloidal stability over time of NP-COOH-TAMRA in different media such as water (black), complete cell medium (DMEM, red) and HEPES buffer (pH 7.4, blue); the standard deviation is visually depicted using a shaded area proportional to the variability in the data.

The effectiveness of the coating procedure was evaluated by several means. Scanning electron microscopy images (SEM) confirm that the morphology and dimensions of the gold core were preserved (Fig. S14†). Also, the optical properties of the particles were preserved, as indicated by superimposable extinction spectra (Fig. 6a).

The hydrodynamic diameters were evaluated with dynamic light scattering (DLS) measurements before and after PEGylation. Results did not show a clear increase upon functionalization. However, this was an expected result considering the relative size of the NPs and the PEG molecule. To confirm the PEGylation we performed inductively coupled plasma optical emission spectroscopy (ICP-OES) and Raman spectroscopy analysis. In ICP-OES analysis, both Au (from the NP) and S (from PEG) were detected on purified PEGylated NP. The obtained values were 14.83 ppm and 0.09 ppm for Au and S, respectively (on the diluted sample, see Methods section). S representing a 0.61% w/w relative to Au. Raman spectroscopy (Fig. S15†) analysis of PEGylated NPs presented the characteristic peak between 250 and 350 cm^−1^ of Au–S stretching vibration, confirming the binding of thiol groups from the PEG chains to the gold nanoparticles.^48^

Subsequently, the PEGylated particles were further functionalized with the fluorescent reporter 5(6)-TAMRA cadaverine (TAMRA) by using a strategy involving carboxyl-to-amine crosslinking. To do this, carboxylic group activators were utilized (see Fig. S16† and the Methods section).

The hydrodynamic diameters were evaluated with dynamic light scattering (DLS) measurements before and after functionalization. The increase of the hydrodynamic diameter in NP-COOH-TAMRA (from 229.1 ± 2.8 nm of bare NPs to 243.6 ± 16.8 nm for NP-COOH-TAMRA) is due to the new organic shell surrounding the particles (Fig. S17 and Table S2†). The ζ-potential values (Fig. 6c and Table S2†) remained around – 20 mV in all cases., *i.e.* similar ζ-potential values of the particles before and after the TAMRA functionalization were obtained. To preserve the colloidal stability of the dispersion, guaranteed by the electrostatic repulsion of the negative –COO^−^ species and the steric hindrance of the PEG shell, the amount of TAMRA dye added was controlled to saturate less than 50% of the particle surface carboxylic groups (see Table S3†). This controlled surface modification ensures that the overall surface charge, dominated by the remaining deprotonated carboxyl groups, remains sufficiently negative to ensure NP dispersion stability. The amount of immobilized TAMRA was quantified by comparing absorption spectra before and after labelling (Fig. S18†), revealing 3 × 10^4^ TAMRA/NP. The presence of the grafted dye onto the particle surface was confirmed by the characteristic emission profile of TAMRA, with a maximum at about 580 nm,^49^ in the emission spectrum collected on the purified NP-COOH-TAMRA particles (Fig. 6b).

The colloidal stability of NP-COOH-TAMRA was studied in different media such as water, complete cell culture medium (Dubelcco's Modified Eagle Medium, supplemented with 10% fetal bovine serum and antibiotics, DMEM) or HEPES buffer up to 7 days (Fig. 6d, Fig. S19 and Table S4†). NP-COOH-TAMRA particles always remained stable in all conditions. As expected, an increase in size was observed for the particles in complete cell media because of non-specific adsorption of proteins.^50^

The molar concentration of NPs was determined by inductively coupled plasma optical emission spectroscopy (ICP-OES). Because of the highly homogeneous NPs produced with this methodology with a standard deviation below 5%, using geometrical considerations and determining the amount of elemental gold represents an accurate method to determine the molar concentration of the NPs from a solution. As an indication of all the parameters considered for molar particle concentration calculation, we reported in Fig. S20† an example. This also enabled the calculation of the particles’ extinction coefficient (*ε*_450 nm_ = 1.47 × 10^11^ M^−1^ cm^−1^ see Fig. S21† and the Methods section for further details).

The functionalization procedure was highly reproducible as stated by extinction spectra and DLS analysis of different batches (Fig. S22 and Table S5†).

**Fig. 7:**
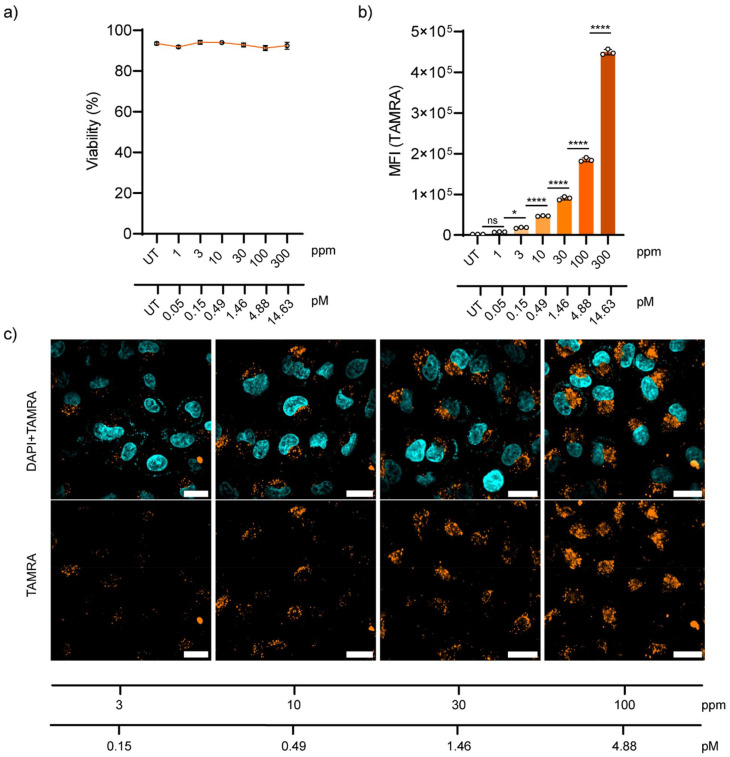
*In vitro* studies in A549 cells. (a) Cell viability assay after the incubation with different concentration of NP-COOH-TAMRA for 24 h, assessed by DRAQ7™ staining (data expressed as mean ± s.d., *n* = 3). UT = untreated. (b) Flow cytometry quantification of the cellular uptake after the incubation with different concentration of NP-COOH-TAMRA for 24 h. Median fluorescence intensity values of TAMRA (median ± s.d., *n* = 3). Statistical analysis was assessed by one-way ANOVA test (ns = non-significant, *p = 0.05, ****p < 0.0001). (c) Representative confocal microscopy images of cellular uptake after the incubation with different concentrations of NP-COOH-TAMRA for 24 h. Top panel: TAMRA fluorescence signal (orange) merged with DAPI fluorescence signal (cyan) for the nuclei staining. Bottom panel: TAMRA fluorescence signal (orange) alone. Scale bars: 20 μm. To ensure reproducibility and reliability of the results, three independent measurements have been conducted on each sample.

Later, the behaviour of NP-COOH-TAMRA in living cells was tested. A549 adenocarcinoma cells were selected as they are a cell line widely used in drug delivery and pharmacological research.^51^ Firstly, the biocompatibility of the NPs was assessed using DRAQ7™ nuclei staining. DRAQ7™ dye is a far-red fluorescent dye that only stains the nuclei of dead cells or cells with compromised membrane integrity, making it ideal for the quantification of nonviable cells.^52^ Cells were incubated with increasing NP concentrations of up to 300 ppm (approximately 14.63 pM of NPs) and then stained with DRAQ7™. Cell viability after 24 h exposure to the NPs is shown in Fig.7a as a percentage of DRAQ7™ negative cells (alive cells) (Fig. S23†). For all tested conditions, the cell integrity was not compromised showing cell viabilities above 90% after 24 h of incubation with the NPs.

The uptake of NP-COOH-TAMRA was studied both by flow cytometry and confocal microscopy. For this aim, A549 cells were incubated with the NPs at final concentrations of 1–3–10–30–100–300 ppm (corresponding to 0.05–0.15–0.49–1.46–4.88–14.63 pM, respectively) for 24 h in complete cell media at 37 °C, 5% CO_2_. Flow cytometry showed the concentration-dependent internalization of NPs (Fig. 7b). Interestingly, it was observed that at concentrations as low as 3 ppm a significant level (*i.e.* 10-fold the control signal) of cellular internalization was measured (Table S6†).

Microscopy images of A549 cells incubated for 24 h with NPs at the selected concentrations confirmed the concentration-dependent NP uptake (Fig. 7c), in agreement with the flow cytometry results. Moreover, confocal microscopy images showed a distribution of the NPs in the perinuclear region of the cells in the form of a punctuated distribution, and it was consistent and homogeneous throughout the sample. This distribution is typical for endocytosed nanomaterials, which is in agreement with the expected behaviour considering the physicochemical properties of our NPs.^53^

## Materials and methods

### Fabrication of the Si master

The replication master mould was fabricated on a 150 mm silicon wafer using e-beam lithography and reactive ion etching for pattern transfer to the substrate. The substrate surface is first cleaned using standard RCA cleaning methods and vapor primed with a hexamethyldisilazane (HMDS) resist adhesion promoter. A chemically amplified high-sensitivity positive-tone photoresist is then spin-coated using a fully automated GAMMA cluster coater-developer tool and the resist film thickness was measured interferometrically using Promicron Nanocalc. After spin-coating, the resist was post-apply-baked and the patterns were exposed using a Vistec SB4050 VSB tool at 50 keV acceleration voltage and 20 A cm^−2^ current density. The resist was then post-exposure-baked and developed using a double puddle process with a 2.38% tetramethylammonium hydroxide (TMAH) based developer. The patterned resist serves as a hard mask for the subsequent pattern transfer to the substrate. This is done by reactive ion etching of the silicon using sulphur hexafluoride (SF_6_) and octafluorocyclobutane (C_4_F_8_) based processes with an STS Pegasus etching tool. After the resist mask was stripped in an ozone (O_3_) plasma, a final RCA-cleaning step followed to remove resist and etching residuals. That way, a nanostructured array of elliptical pillars with an area size of 1 × 1 cm^2^ and ellipse dimensions of 400 nm in length and 200 nm in width was patterned. The size of the unit cell was 600 × 400 nm^2^ resulting in an interpillar distance of 200 nm. The height of the pillars was 255 nm. For process and quality control, the structures produced on the final master mould were inspected using top-view SEM imaging with a LEO 1560 SEM tool. For profile and etch depth analysis, both SEM cross-section and profilometric measurements with a Veeco Dektak 8 were also carried out on preliminary test wafers.

### Fabrication of the intermediate imprint

The custom silicon UV-NIL master was treated with an anti-sticking layer (BGL-GZ-83, PROFACTOR GmbH, Steyr, Austria). The anti-sticking reagent was first filtered through a syringe filter (200 nm pore PTFE membrane filter from Acrodisc® purchased from VWR International, Radnor, USA). 2 mL of the reagent were placed in the middle of the Si master wafer by drop casting from a beaker glass and the spin coating process was started (1000 rpm for 30 s followed by 2000 rpm for 30 s). For the intermediate imprint, we used fresh glass wafers (polished fused quartz wafers, Siegert Wafer GmbH, Aachen, Germany) which were cleaned on the spin coater (M-SPIN 150, Ramgraber, Hofolding bei Brunnthal, Germany) by splashing acetone onto the rotating wafer immediately followed by isopropanol (200 rpm for 5 s for each solvent). This was followed by 30 s at 2000 rpm to remove all the solvent. The now dry wafer was placed on a hotplate (Präzitherm PZ28-2, Harry Gestigkeit GmbH, Duesseldorf, Germany) for 20 min at 170 °C to remove all potential water residues. After cool-down, we coated the glass wafer with an adhesion promotion layer (OrmoPrime08, micro resist technology GmbH, Berlin, Germany) by a single step spin coating process. Specifically, 4 mL of adhesion promoter reagent were placed in the middle of the wafer by drop casting from a beaker glass and the spin coating process was started (4000 rpm for 60s). The adhesion promotion layer was activated on a hotplate at 150 °C for 5 min and then the wafer cooled down to room temperature. The intermediate imprint was done by placing a drop of UV curable resist (OrmoComp®, micro resist technology GmbH, Berlin, Germany) onto the Si master directly next to the nanostructured area. Then the glass wafer was placed on top of the Si master wafer by letting it tip over the drop of resist so that it was automatically pressed into the nanostructured region to be replicated. Air bubbles disappeared automatically after waiting for 10–15 minutes. The stack of the two wafers was treated by UV light flood exposure (nailstar® Professional, London, UK) at a power density of 4 mW cm^−2^ at 385 nm for 10 min to cure the resist in between the two wafers. Afterwards, the two wafers were separated manually in a single movement at constant applied force and, as a result, a negative image of the nanostructure formed in the UV-curable resist was obtained on the glass wafer.

### Fabrication of the PDMS stamp

From the intermediate imprint, a hybrid stamp composed of soft polydimethylsiloxane (s-PDMS) and hard PDMS (h-PDMS) was fabricated. In a preparatory step, the glass wafer with the intermediate imprint was treated with an anti-sticking layer as described above for the Si master. The fabrication of the hybrid stamp followed methods reported in the literature.^40,54,55^ Briefly, for the fabrication of the h-PDMS layer, 3.4 g of PDMS prepolymer (VDT-731, Gelest Corp., Morrisville, USA) were mixed with 18 μL of platinum catalyst (SIP6831.2, Gelest Corp., Morrisville, USA) and 5 μL of modulator 1,3,5,7-tetravinyl-1,3,5,7-tetramethylcyclotetrasiloxane (SIT7900.0, Gelest C.) and degassed in a desiccator until no air bubbles were visible. Then, 1 g of hydrosilane prepolymer (HMS-301, Gelest Corp., Morrisville, USA) was added, the solution gently stirred, and then again degassed in a desiccator until no air bubbles were visible. The h-PDMS solution was poured onto the intermediate imprint and degassed again in a desiccator. Afterwards, the wafer was placed on the spin coater and spinned for 30 s at 3000 rpm to create an even layer of h-PDMS. The so-coated wafer was placed on a hotplate at 50 °C for 5 min. Then, a 1–2 mm thick layer of s-PDMS was fabricated on top of the h-PDMS. Here, we used a standard PDMS that was prepared according to the manufacturers’ datasheet by mixing 10 parts of the base elastomer with 1 part of the curing agent (Sylgard 184 purchased from Farnell GmbH, Poing, Germany) and degassed in a desiccator for 45 min. The so-prepared sample was then placed on a hotplate at 50 °C for 12 h to finish the curing of the PDMS layers. In a final step, the hybrid h-PDMS/s-PDMS layer was separated manually from the glass wafer in a single movement at constant applied force. As a result, a negative image of the nanostructure from the intermediate imprint was obtained on the h-PDMS/s-PDMS, which served as stamp for the nanostructure replication by UV-NIL.

### Nanostructure replication by UV-NIL and fabrication of nanoparticle dispersions

Fresh Si wafers (polished 4′′ Si wafers purchased from Active Business Company GmbH, Marquartstein, Germany) were cleaned on the spin coater by splashing acetone onto the rotating wafer immediately followed by isopropanol (200 rpm for 5 s for each solvent). This was followed by 30 s at 2000 rpm to remove all solvent. The now dry wafer was placed on a hotplate for 20 min at 170 °C to remove all potential water residues. After cool-down, the adhesion promotion layer was spin-coated onto the wafer. The adhesion promoter (HMNP12, PROFACTOR GmbH, Steyr-Gleink, Austria) was first filtered through a syringe filter. 2 mL of the adhesion promoter reagent were placed in the middle of the wafer by drop casting from a beaker glass and the spin coating process was started (500 rpm for 5 s followed by 3000 rpm for about 15 s). Best results were achieved when stopping the spinning once no visible change of colour was observed. The adhesion promotion layer was activated on a hotplate at 120 °C for 1 min and then the wafer cooled down to room temperature. In a next step, the lift-off resist (LOR1A, micro resist technology GmbH, Berlin, Germany) layer was spin-coated onto the wafer following the same procedure as for the adhesion layer with adjusted spin coating process parameters (500 rpm for 3 s followed by 2500 rpm for about 15 s). Again, for best results, the spin coating was stopped once no colour change was visible anymore. Afterwards, a soft bake was done on a hotplate at 150 °C for 5 min. As a final layer we deposited the NIL resist (mr-NIL212-FC-200 nm, micro resist technology GmbH, Berlin, Germany). The same procedure as for the previous layers was applied with adjusted spin coating process parameters (500 rpm for 5s followed by 4000 rpm for about 15 s). The soft bake for the NIL resist was done at 100 °C for 1 min on a hotplate. On these wafers the NIL process was carried out using a nanoimprint lithography step and repeat tool (Soft-NIL-Stepper C-1-2-N, PROFACTOR GmbH, Steyr-Gleink, Austria) by pressing the hybrid h-PDMS/s-PDMS stamp into the resist layers and curing the latter by UV light. The h-PDMS/s-PDMS stamp was assembled on a PDMS membrane according to the instructions given in the manual of the nanoimprint lithography step and repeat tool. Multiple imprints on a single wafer were performed (see Fig. S3† for a photograph of a substrate with 28 individual imprints). For each individual imprint, the UV light exposure was done for 5 s at a power density of 146 mW cm^−2^ at a wavelength of 365 nm. Afterwards, the imprinted wafer was treated by UV light flood exposure for 3 min at a power density of 4 mW cm^−2^ to cure the resist in between the individual imprints.

The dry-physical etching step was done by Ar ion etching in a plasma oven equipped with a reactive ion etching electrode (FEMTO RIE, Diener Electronic GmbH operating at 13.56 MHz, Ebhausen, Germany). To achieve best possible anisotropic etching perpendicular to the wafer surface, we conducted the Ar plasma etching step at a low pressure of 0.04 mbar with a power of 50 W for 5.5 min. The Ar gas flow was adjusted to 1 sccm. The Ar etching process removed the residual layer and the lift-off resist inside the elliptical holes in the nanostructured area. A quick wet-chemical etching process was done by a developer solution (MICROPOSIT MF-24A, micro resist technology GmbH, Berlin, Germany) for 3 s to etch the lift-off resist, which created the undercut etching structure. The wafer was rinsed thoroughly with ultra–pure water (18.2 MΩ cm resistivity).

The sacrificial aluminium-doped zinc oxide (AZO) layer was deposited by DC magnetron sputtering (UNIVEX 450C, Leybold GmbH, Cologne, Germany) with a target to substrate distance of about 100 mm. AZO was sputtered in pure Ar atmosphere at a gas pressure of 2 μbar and at a power of 80 W with a sputter rate of 0.324 nm s^−1^. The gold layer was applied by a thermal vacuum evaporation process in a vacuum better than 10^−6^ mbar and at a deposition rate of 0.03 nm s^−1^ (UNIVEX 450 thermal evaporation system, Leybold GmbH, Cologne, Germany).

After the deposition of the gold layer, a lift-off process was done in which the lift-off resist was removed in a wet-chemical etching step by immersion of the wafer in the developer solution (the same as mentioned above). The process was stopped after removal of all the lift-off resist by immersion of the wafer in ultra-pure water. The wafer was then rinsed thoroughly with water and blown dry with nitrogen. The result was an array of NPs on the wafer surface.

The NPs were transferred from the wafer substrate to solution by immersion of the wafer in the developer solution for at least one hour in an ultrasonic bath (USC 600 TH operated at 120 W, VWR International, Radnor, USA). The obtained NP dispersion was diluted with ultra-pure water and washed at least three times. Washing can be done either by centrifugation or by using gravitation and letting the NPs sediment for 12 h and removal of the supernatant by a pipette.

### Characterization of the nanostructures and the nanoparticle dispersions

Scanning electron microscopy (SEM) images were captured on a Zeiss Supra 40 electron microscope at 5 kV acceleration voltage using the in-lens detector. The nanostructures directly after imprint and the array of NPs were imaged directly on the substrate. The images of the intermediate imprint and the hybrid h-PDMS/s-PDMS stamp were taken after sputter deposition of a 20 nm thick Pd layer for the generation of a conductive top layer to allow for SEM imaging (Cressington 108auto sputter coater, Dortmund, Germany). The NPs from aqueous solution were prepared for imaging by drop-casting onto a Si wafer substrate with a native oxide surface and letting the solution dry at room temperature. SEM-EDX images were acquired by an EDAX Octane Elect Plus EDX system and APEX Standard software.

Nanoparticle geometry was analysed based on SEM images. Here, 12 batches of NPs across all fabricated thicknesses were investigated for the calculation of the mean values and the standard deviations of the width and the length of the elliptical platelet NPs. The AZO sample surface topography was characterized by scanning force microscopy (SFM) with an instrument (Molecular Imaging, Pico Plus) operated in acoustic mode with SSS-NCHR-10 tips (NANOSENSORS SuperSharpSilicon™ – SPM Sensor, Neuchatel, Switzerland). SFM images were analysed and plotted using the Gwyddion software. The software is available as free software under the terms of the GNU General Public License.

Optical absorbance of the obtained NP dispersions was measured on two instruments, which are a UV Vis Spectrophotometer (Nicolet evolution 100, Thermo Scientific, Waltham, USA) and a Fourier transform infrared spectrometer (Bruker Vertex 70, Bruker Corporation, Billerica, USA) equipped with an additional visible light source and a Si photodetector. The NP dispersions were examined in a PMMA cuvette (semi-micro PMMA cuvette, BRAND GMBH + CO KG, Wertheim, Germany) with 1 cm optical path length. The measurements were recorded at an incident light beam normal to the cuvette surface and referenced to a cuvette filled with water. The obtained absorbance curves were smoothed and plotted using commercially available software (OriginLab Corporation, Northampton, USA).

### Simulation of the optical nanoparticle properties

For the simulation of the optical extinction spectra of the different NPs, we employed the discrete dipole approximation method using the freely available software DDSCAT7.3.^42^ The simulation targets were created using the code embedded in the DDSCAT software and OriginLab. NP orientations and light polarization directions were set according to the functionalities provided by the simulation software. The calculations were carried out using the wavelength-dependent complex refractive index values of Au by Johnson and Christy.^56^ The NPs were simulated with water as surrounding medium with a real refractive index of 1.33. The obtained extinction curves were plotted using the OriginLab software.

### Materials for nanoparticle surface modification

***All the materials including α-Carboxy-ω-Thiol poly(ethylene glycol) (SH-PEG-COOH, *M*_n_ 5000, Rapp Polymere GmbH), Tetramethylrhodamine-amine 5 isomer (TAMRA, 95%, Lumiprobe GmbH), *N*-(3-(Dimethylamino)propyl)-*N*′-ethylcarbodiimide hydrochloride (EDC, crystalline, Sigma-Aldrich), *N*-hydroxysulfosuccinimide sodium salt (sulfo-NHS, >98%, Sigma Aldrich), 4-(2-hydroxyethyl)-1-piperazineethanesulfonic acid (HEPES, ≥99.5%, Sigma-Aldrich), sodium hydroxide (NaOH, ≥97.0%, Sigma-Aldrich), Dulbecco's Modified Eagle Medium (DMEM Gibco, Thermo Fisher Scientific, Massachusetts, USA) were used as purchased without any purification. Ultrapure water (≤15.0 MΩ) produced by a Millipore Milli-Q gradient system from Merck was used as solvent.

### Characterization of the nanoparticle surface modifications

#### UV-Vis/fluorescence spectroscopy

A Cary 3500 Multicell Peltier UV-Vis Spectrophotometer was used to record the UV-vis extinction and absorption spectra of samples in aqueous solution. Fluorescence characterization of NP-COOH-TAMRA aqueous suspension was performed using an Infinite® 200 PRO Plate reader, Tecan, Switzerland.

#### Inductively coupled plasma-optical emission spectrometry (ICP-OES)

Elemental analysis was conducted using an Agilent 5800 Inductively Coupled Plasma Optical Emission Spectrometer. Before analysis, the samples were subjected to acid digestion overnight using a mixture of aqua regia (HCl/HNO_3_ 3 : 1). For gold (Au) detection, concentrations were determined using two atomic emission lines at 242.794 nm and 267.594 nm. To demonstrate the presence of PEG chains on the NP surface, sulphur (S) detection was also carried out by collecting emission lines at 180.669 nm and 181.972 nm; a dilution factor of 1 : 250 was applied to ensure the elemental concentrations of the sample fell within the calibration range. Calibration curves were prepared with concentrations ranging from 0 to 10 ppm.

#### Calculation of the particles’ molar extinction coefficient

Once evaluated the started concentration of NP-COOH by means of ICP-OES (see Fig. S20† for more details), known dilutions of the starting solution were produced and analysed by UV-Vis spectroscopy, ensuring that the absorption value remained below 1 OD (optical density). After collecting the spectra, the absorption values at 450 nm were plotted against the concentration of the particles (expressed in molarity [M]). Through linear fitting, the mathematical equation of the line and the *R*^2^ coefficient were determined. Since the intercept was approximately zero, the slope (1.47 × 10^11^ M^−1^ cm^−1^) of the curve represents the molar extinction coefficient (*ε*_450 nm_) of the particles.

#### Dynamic light scattering

The hydrodynamic diameter (*D*_h_), polydispersity index (PDI) and zeta potential (ζ-potential) of the NP samples were derived through dynamic light scattering (DLS) using a Malvern Zetasizer Nano ZSP equipped with a 10 mW He–Ne laser (wavelength 633 nm) and a fixed scattering angle of 173°.

#### Raman spectroscopy

Raman spectrum was acquired using a Renishaw inVia Reflex system on a glass substrate, employing a 785 nm laser and a 50× objective lens. The samples were exposed to 30 mW laser power, with an acquisition time of 15 seconds per scan and 3 accumulations.

#### Colloidal stability tests

DLS and UV-Vis measurements were carried out at different times (up to one week) in different media: MilliQ water, complete cell culture medium (DMEM: Dulbecco's Modified Eagle Medium without phenol red (DMEM; Gibco)) supplemented with 10% foetal bovine serum (FBS; Gibco) and 1% Penicillin–Streptomycin (P–S; Invitrogen) and HEPES buffer (pH 7.4, 50 mM, Gibco).

### PEGylation of nanoparticles

Before performing any reaction, all the glassware was cleaned with aqua regia (HCl/HNO_3_ 3 : 1 v/v).

The NPs were preliminary washed twice with water by centrifugation (5000*g*, 2 min). PEG (SH-PEG-COOH, molecular weight of 5 kDa) was first dissolved in water in a concentration of 10 mg mL^−1^. Then, in a vial, equipped with a magnetic stirrer (500 rpm), 1.0 mL of NP sample (conc. 40 pM) was mixed with 100 μL of the aqueous PEG solution and 11.0 μL of NaOH solution (2 M). The system was kept under stirring (500 rpm) overnight.

The purification procedure was performed by centrifugation (5000*g*, 2 min) washing the particles 3-times with water. The final particles were suspended in 1 mL of water.

### TAMRA labelling

In a 2 mL vial, equipped with a magnetic stirrer (500 rpm), 500 μL of NP-COOH (12 pM) were first mixed with the carboxylic acid activators, *i.e.* 1.2 μL of EDC (aqueous solution 1 mg mL^−1^) and 1.2 μL of sulfo-NHS (aqueous solution 2.7 mg mL^−1^) at pH 5 (MES buffer 50 mM); after 20 minutes, 25.1 μL of TAMRA-amine (aqueous solution 14 μM) were added to the reaction mixture. After additional 15 minutes, the pH was increased through the addition of 1.5 μL of NaOH (2 M) to stop the reaction.

The system was kept under stirring overnight in dark condition. Then, purification steps were performed by means of centrifugation (5000*g*, 2 minutes), by alternating the use of water/methanol (50%) and water (at least 6 washes in total). The washing steps were reiterated until the supernatant presented a fluorescence signal <10% of the fluorescence of the particles.

Then, the purified sample was suspended in 500 μL of water.

### *In vitro* study

#### Cell culture

Adenocarcinoma human alveolar basal epithelial cells (A549 cells, ATCC®) were cultured in Dulbecco's Modified Eagle Medium (DMEM, high glucose (4.5 g L^−1^) and pyruvate, Gibco), supplemented with 10% foetal bovine serum (FBS, Gibco) and Penicillin–Streptomycin (P/S, 50 U mL^−1^–50 μg mL^−1^) (Gibco) in a humidified chamber at 37 °C under 5% CO_2_. Cells were passaged after cleaning Dulbecco's Phosphate Buffered Saline (PBS, 1×) (Gibco) with 0.25% Trypsin-EDTA when the culture reached confluency.

#### Uptake studies

##### Flow cytometry

Cells were seeded on a 96-well plate at a density of 7500 cells per well in 0.1 mL of complete media. After 24 h, the cells were washed with PBS 1× and the medium was replaced with the freshly prepared NP dispersions, diluted in complete media. Uptake experiments were performed by exposing the cells to different NP-COOH-TAMRA concentrations (300–100–30–10–3–1 ppm) at 37 °C and 5% CO_2_ for 24 h. After NP exposure, the cells were washed twice with 0.1 mL PBS 1× per well. Then, the cells were harvested after trypsinization for 2 min with 50 μL 0.25% Trypsin–EDTA. 0.15 mL of PBS 1× supplemented with 2% of FBS was added to each well to recover the cells. The cells were transferred to a V-bottom 96 well plate and pelleted by centrifugation at 500*g* for 3 min. The cells were finally resuspended in 200 μL of PBS 1× with 2% of FBS and analysed by flow cytometry (Beckman Coulter Cytoflex S B-R-V-Y Series, C02947) using a 561 nm excitation and measuring fluorescence emission of TAMRA (*i.e.*, 610/20 bandpass). Results were analysed using BD FlowJo 10.10.0. and reported as the median of cell fluorescence intensity.

##### Confocal microscopy

Cells were seeded in a μ-Slide 18 well-ibiTreat chamber (0.34 cm^2^ per well, Ibidi, Germany) at the concentration of 7500 cells per well. After 24 h, the cells were washed with PBS 1× and the medium was replaced with the freshly prepared NP dispersions, diluted in complete media. Uptake experiments were performed by exposing the cells to different NP-COOH-TAMRA concentrations (100–30–10–3 ppm) at 37 °C and 5% CO_2_ for 24 h. After NP exposure, the cells were washed twice with fresh PBS 1× to remove those NPs that were not associated to cells and incubated with 3.6% formaldehyde solution (Sigma Aldrich) in PBS 1× solution for 20–25 min at room temperature. Then, the formaldehyde solution was removed, and cells were washed with PBS 1× before adding a few drops of Ibidi Mounting Medium with DAPI (Ibidi, Germany). Confocal images were captured on an Andor Dragonfly spinning disk confocal system mounted on a Nikon TiE microscope equipped with a Zyla 4.2 PLUS camera (Andor, Oxford Instruments). For NP detection, TAMRA was excited with a 561 nm laser and the emitted fluorescence was collected by a 620 (50) nm filter. For the nuclei detection, DAPI was excited with a 561 nm laser and the emitted fluorescence was collected by a 450 (50) nm filter. Images were taken with a 60× objective. All the images were processed with ImageJ®.

#### Cell viability assay

To study the number of viable cells after exposure to the NPs, DRAQ7™ (Thermo Fisher) was used. DRAQ7™ is a far-red viability dye used for investigating dead or membrane compromised cells, notably for dead cell quantification in flow cytometry. A549 cells were seeded on a 96 well plate at density of 7500 cells per well in 0.1 mL of complete media. After 24 h, the cells were washed with PBS 1× and the medium was replaced with the freshly prepared NP dispersions, diluted in complete media at 37 °C and 5% CO_2_ for 24 h. After NP exposure, the cells were washed twice with 0.1 mL PBS 1× per well. Then, the cells were harvested after trypsinization for 2 min with 50 μL 0.25% Trypsin-EDTA. 0.15 mL of PBS 1× supplemented with 2% of FBS was added to each well to recover the cells. The cells were transferred to a V-bottom 96 well plate and pelleted by centrifugation at 500 g for 3 min. The cells were finally resuspended in 200 μL of PBS 1× with 2% of FBS at 0.75 μM DRAQ7™. The cells were incubated for 10 minutes at room temperature and protected from light. Afterwards, the stained cells were analysed by flow cytometry using excitation at 638 nm and by measuring fluorescence emission of DRAQ7™ (*i.e.*, 690/50 bandpass). Results were analysed using BD FlowJo 10.10.0. and the viability was reported as the percentage of non-stained cells (Fig. S23†).

## Conclusions

In this work, we have proven the usefulness of the NIL technique in conjunction with thin film deposition methods to produce plasmonic gold NPs with distinct optical properties in the near-infrared region of light. This fabrication technology together with the demonstrated simulations of the optical properties allows the selection of optimum NP geometries with tailor-made optical properties for specific applications. Due to the versatility of thin film deposition using thermal evaporation or sputter deposition, our method can be easily applied to other materials than gold and also layers of different materials, such as magnetic materials, or luminescent materials. The fabrication method renders highly homogenous NPs with size standard deviations below 5%. Our NP fabrication process can be easily scaled up by using roll-to-roll or roll-to-plate NIL techniques, which are already established in industry.

Moreover, we have demonstrated that these NPs are susceptible to be further functionalized by applying a PEGylation protocol used for materials synthesized by wet chemistry methods. The NIL and PEGylation process were highly reproducible providing batches with homogeneous physicochemical characteristics. Subsequently, PEGylated NPs were further modified with a fluorescence reporter (TAMRA) and showed great colloidal stability even in complex biological media such as complete cell media (DMEM). Finally, these NPs showed good biocompatibility in living cancer cells and a dose-dependent internalization rate.

In the future, these NPs could potentially replace conventional nanomaterials in biomedical applications that utilize light at specific wavelengths. The key prerequisites for their regular use in biomedical applications are the reproducibility and reliability of the NP fabrication, along with defined optical responses and low nanotoxicity. Potential applications include using these NPs as contrast agents for imaging, as probes for targeted photothermal cancer therapy, and as probes for sensing applications based on optical detection methods.

## Author contributions

Pauline Kolar-Hofer: investigation, validation, visualization, writing – original draft, writing – review & editing. Giulia Zampini: investigation, validation, writing – original draft, writing – review & editing. Christian Georg Derntl: investigation, validation, writing – review & editing. Enrica Soprano: investigation, validation, writing – original draft. Ester Polo: investigation, validation, writing – review & editing. Pablo del Pino: funding acquisition, investigation, supervision, validation. Nurgul Kereyeva: investigation, validation. Moritz Eggeling: investigation, validation, visualization. Leoni Breth: investigation, validation, writing – original draft. Michael J. Haslinger: investigation, validation, writing – review & editing. Michael Mühlberger: investigation, validation, writing – review & editing. Peter Ertl: investigation, validation, writing – review & editing. Astrit Shoshi: investigation, validation, visualization, writing – original draft. Julian Hartbaum: investigation, validation. Michael Jurisch: investigation, validation. Beatriz Pelaz: conceptualization, funding acquisition, investigation, methodology, supervision, validation, visualization, writing – original draft, writing – review & editing. Stefan Schrittwieser: conceptualization, funding acquisition, investigation, methodology, supervision, validation, visualization, writing – original draft, writing – review & editing.

## Data availability

The data supporting this article have been included as part of the ESI.†

## Conflicts of interest

There are no conflicts to declare.

## Supplementary Material

NR-017-D4NR02677B-s001
