# Supplementary material for: Fabrication of nanoparticles with precisely controllable plasmonic properties as tools for biomedical applications
Source: Nanoscale. 2025 Jan 15;17(8):4423–38. doi: 10.1039/d4nr02677b (PMC11734588; doi:10.1039/d4nr02677b)
Supplement: NR-017-D4NR02677B-s001 [file NR-017-D4NR02677B-s001.pdf]

Supplementary Information (SI) for Nanoscale.
This journal is © The Royal Society of Chemistry 2024

Fabrication of nanoparticles with precisely controllable plasmonic properties as tools for biomedical applications – Supporting information

Pauline Kolar-Hofer,[a] Giulia Zampini,[b] Christian Georg Derntl,[a] Enrica Soprano,[b] Ester Polo,[c] Pablo del Pino,[d] Nurgul Kereyeva,[a] Moritz Eggeling,[a] Leoni Breth,[e] Michael J. Haslinger,[f] Michael Mühlberger,[f] Peter Ertl,[g] Astrit Shoshi,[h] Julian Hartbaum,[h] Michael Jurisch,[h] Beatriz Pelaz*[i] and Stefan Schrittwieser*[a]

[a] AIT Austrian Institute of Technology, Molecular Diagnostics, 1210 Vienna, Austria;
[b] Centro Singular de Investigación en Química Biolóxica e Materiais Moleculares (CiQUS), Universidade de Santiago de Compostela, 15782 Santiago de Compostela, Spain
[c] Centro Singular de Investigación en Química Biolóxica e Materiais Moleculares (CiQUS), Departamento de Bioquímica, Universidade de Santiago de Compostela, 15782 Santiago de Compostela, Spain;
[d] Centro Singular de Investigación en Química Biolóxica e Materiais Moleculares (CiQUS), Departamento de Física de Partículas, Universidade de Santiago de Compostela, 15782 Santiago de Compostela, Spain
[e] Department for Integrated Sensor Systems, University for Continuing Education Krems, 2700 Wr. Neustadt, Austria
[f] PROFACTOR GmbH, Im Stadtgut D1, 4407 Steyr-Gleink, Austria
[g] Institute of Applied Synthetic Chemistry, Institute of Chemical Technologies and Analytics, Technische Universitaet Wien (TUW), Vienna, Austria
[h] Institut für Mikroelektronik Stuttgart (IMS CHIPS), Allmandring 30a, 70569 Stuttgart, Germany
[i] Centro Singular de Investigación en Química Biolóxica e Materiais Moleculares (CiQUS), Departamento de Química Inorgánica, Universidade de Santiago de Compostela, 15782 Santiago de Compostela, Spain

Correspondence: beatriz.pelaz@usc.es, stefan.schrittwieser@ait.ac.at

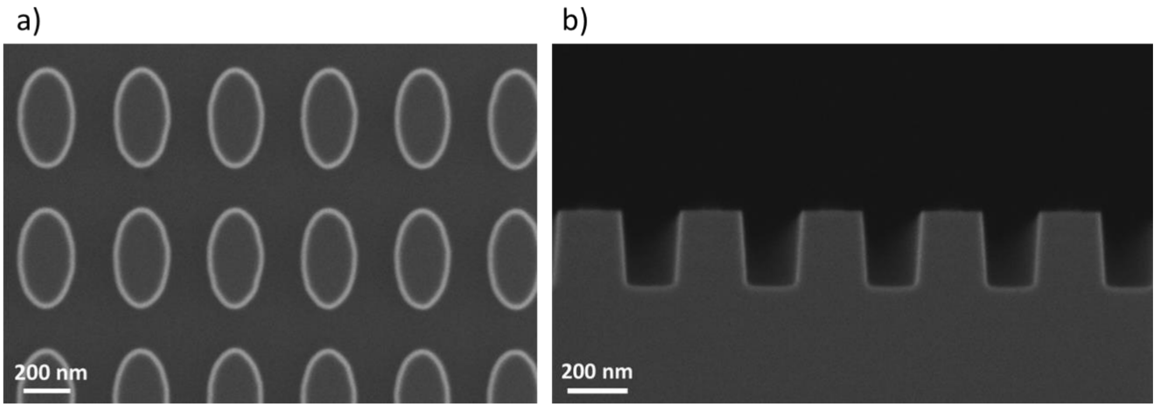

Fig. S1: a) Top-view SEM image of the final Si-master mould consisting of elliptical shaped pillars with semi-minor and semi-major axes of 205 nm x 398 nm, respectively. b) Cross-section SEM image of the preliminary test wafer after resist strip by O_3 plasma showing elliptical pillars with a side wall angle of 87.6° and a height of 255 nm.

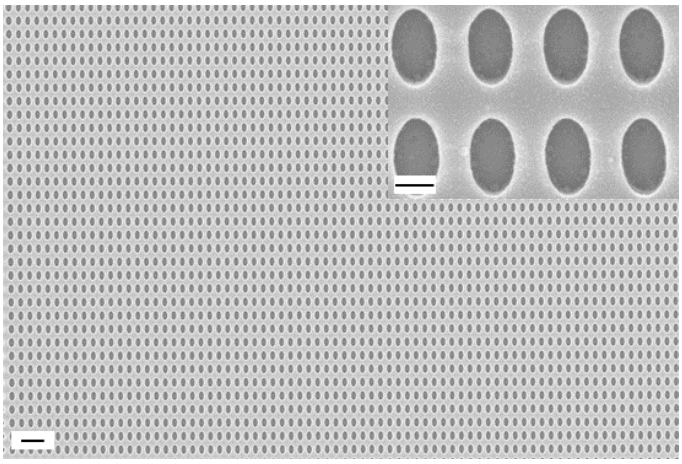

Fig. S2: SEM images of the intermediate replication in OrmoComp resist. Overview at lower magnification and detailed image at higher magnification (inset in the top right). The scale bars correspond to 1 µm for the large overview images and to 200 nm for the insets showing details at higher magnification.

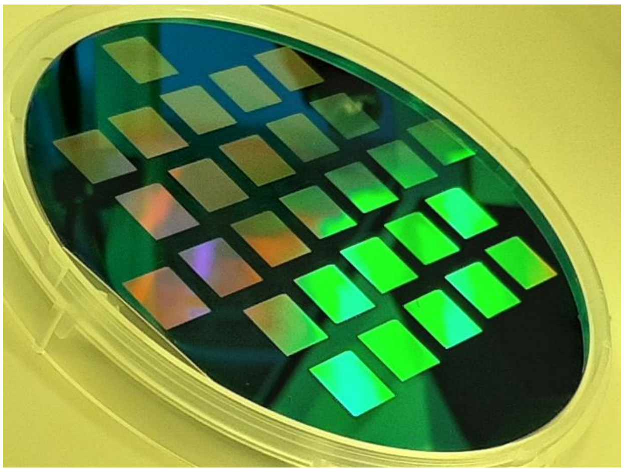

Fig. S3: Photography of the nanoimprinted Si wafer directly after UV-NIL. 28 replicas of the PDMS stamp were fabricated on a Si wafer with 100 mm diameter.

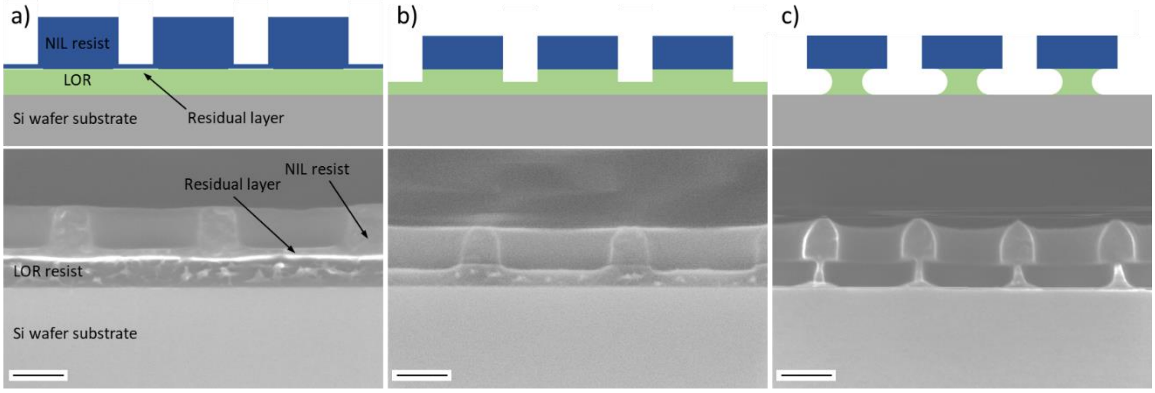

Fig. S4: Sketch and SEM cross sections of imprinted nanostructures. a) Directly after the nanoimprint lithography step. The imprinted NIL resist and the residual layer are on top of the lift-off resist (LOR) layer and the Si wafer substrate b) After the Ar plasma etching step, which removes the residual layer and etches the LOR and NIL resist. c) After the LOR development step an undercut is etched in the LOR. The scale bar corresponds to 200 nm in all images.

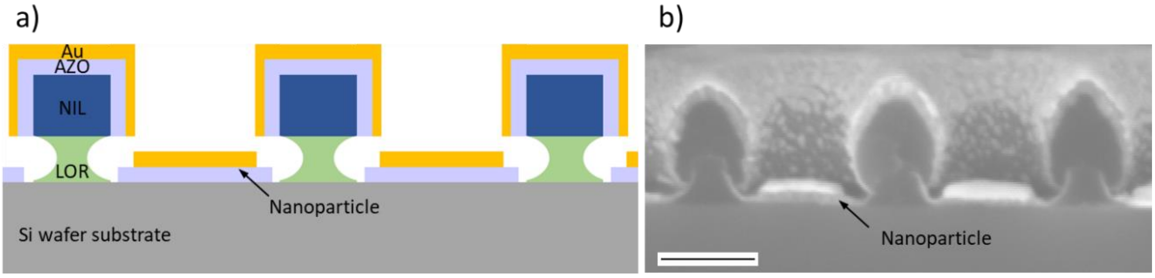

Fig. S5: Sketch and SEM of the cross section of the imprinted nanostructure after the deposition of the sacrificial layer and the gold metal layer. a) Schematic sketch showing the deposited sacrificial aluminium-doped zinc oxide (AZO) and gold metal layer onto the nanostructures after undercut etching of the LOR. The nanoparticles are formed directly on the Si wafer substrate in the elliptical holes in the LOR and NIL resist layers acting as shadow mask. b) SEM image of the nanostructure after the deposition of 25 nm of AZO and 30 nm of gold metal with the fabricated nanoparticles at the bottom of the elliptical holes. The scale bar corresponds to 200 nm.

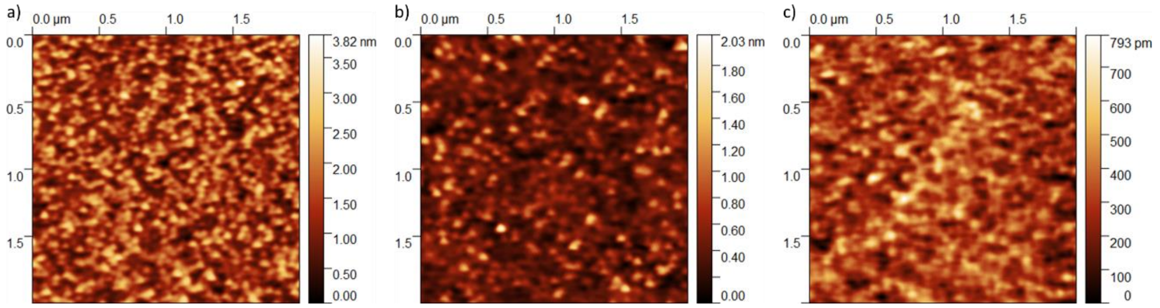

Fig. S6: Scanning force microscopy (SFM) images of a sputtered AZO layer. a) 40 nm thick AZO layer with a measured mean root square roughness of 549 pm. b) 25 nm thick AZO layer with a measured mean root square roughness of 224 pm. c) 10 nm thick AZO layer with a measured mean root square roughness of 104 pm.

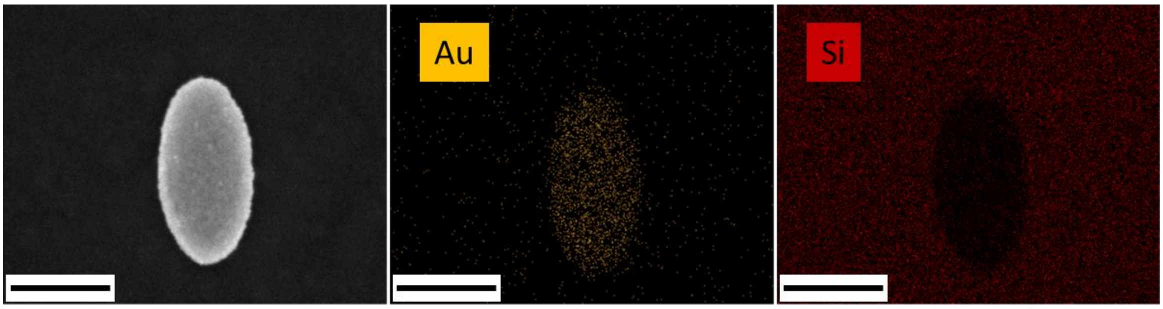

Fig. S7: SEM-EDX analysis of a NP. The gold NP (orange) can be clearly discriminated form the Si substrate (red). The scale bar corresponds to 200 nm.

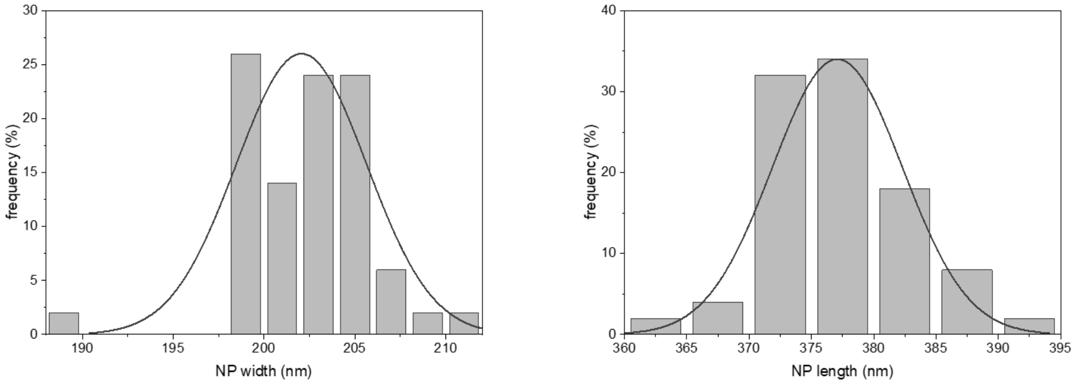

Fig. S8: Histograms and Gaussian fits of the nanoparticle width and length for a nanoparticle thickness of 30 nm, resulting in a mean nanoparticle width of 202.1 ± 3.6 nm and a mean nanoparticle length of 377.1 ± 5.2 nm.

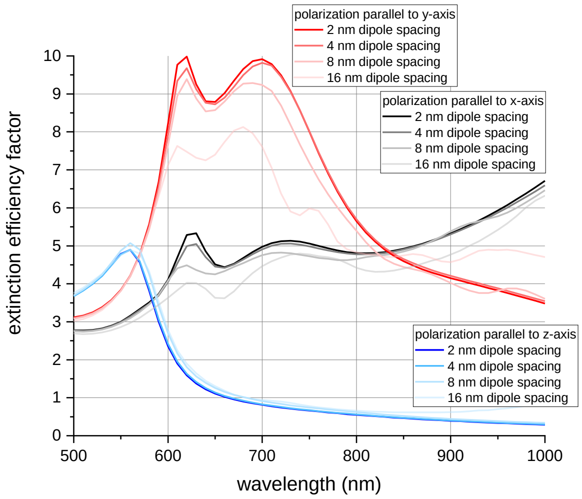

Fig. S9: Convergence study of the simulation model. Three different nanoparticle orientations with respect to the direction of the linear polarization of the excitation light (red curve for a polarization along the long ellipse axis, black for a polarization along the short ellipse axis, blue for a polarization along the nanoparticle thickness). Different dipole spacings are indicated by increasing brightness of the plotted lines for increasing dipole spacing.

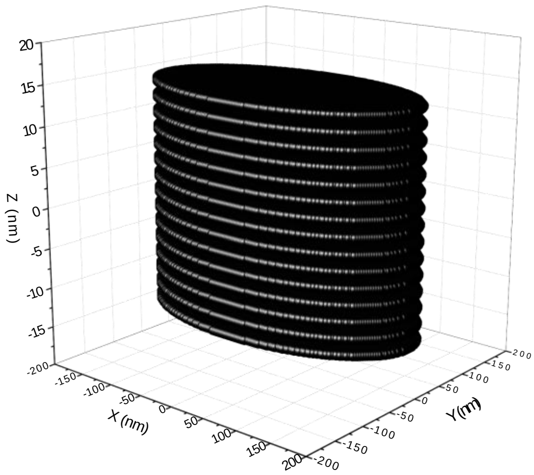

Fig. S10: Dipoles and geometry of a nanoparticle with 30 nm thickness serving as target input file for the DDA simulations with a dipole spacing of 2 nm.

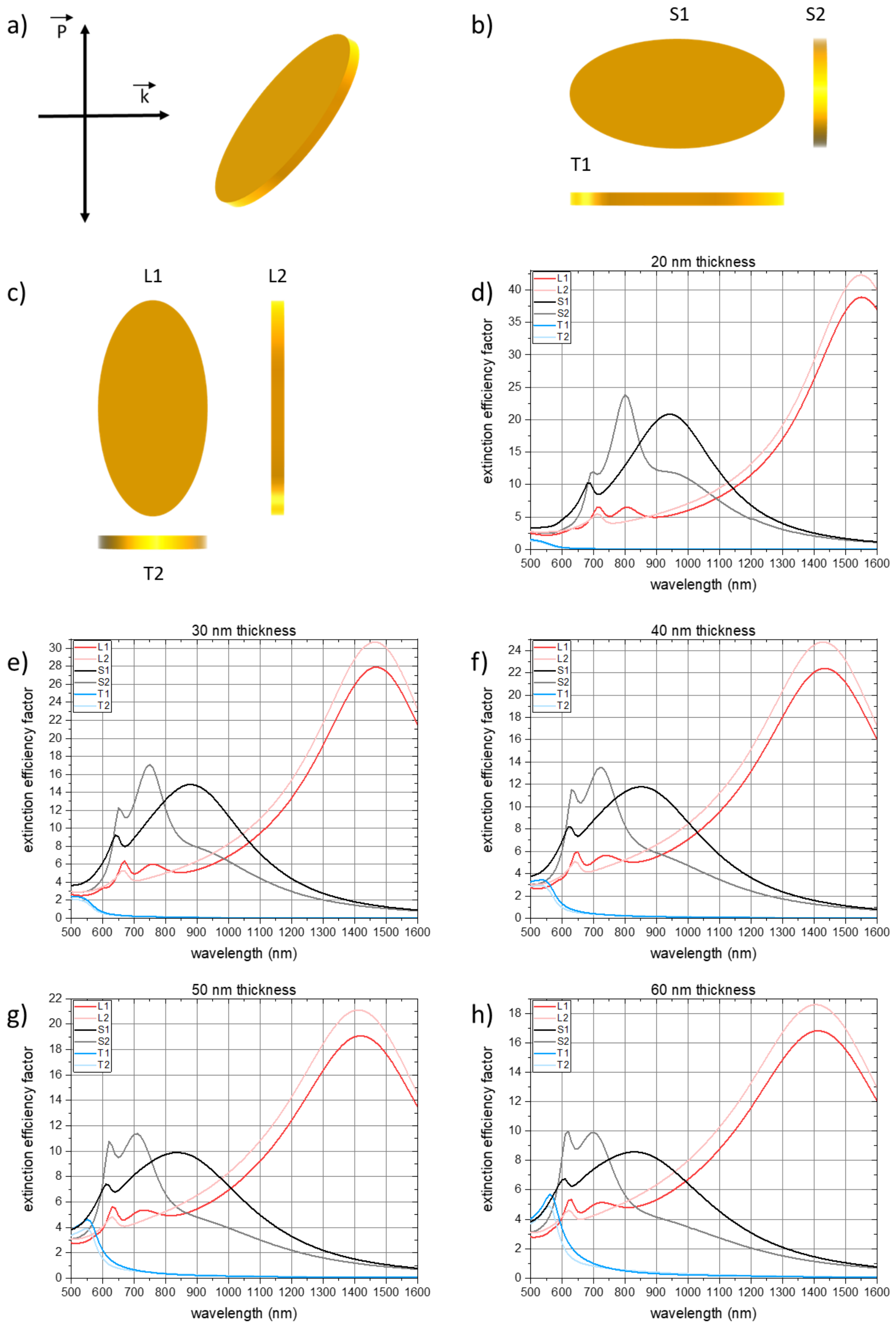

Fig. S11: Simulated extinction efficiency factor for all nanoparticle thicknesses under excitation of linearly polarized light. The extinction efficiency factor for the main nanoparticle orientations with respect to the polarization direction is shown in dependence of the wavelength. a) schematic illustration of the polarization direction vector P and the light propagation vector k. b) schematic nanoparticle orientation with polarization direction along the short ellipse axis (S1 and S2) and first possible orientation towards the nanoparticle platelet thickness (T1). c) schematic nanoparticle orientation with polarization direction along the long ellipse axis (L1 and L2) and second possible

orientation towards the nanoparticle platelet thickness (T2). Simulated extinction efficiency factor for: d) 20 nm thickness; e) 30 nm thickness; f) 40 nm thickness; g) 50 nm thickness; h) 60 nm thickness. S1 and S2 are plotted in black and grey, respectively. L1 and L2 are plotted in dark and bright red, respectively, while T1 and T2 are shown in dark and bright blue.

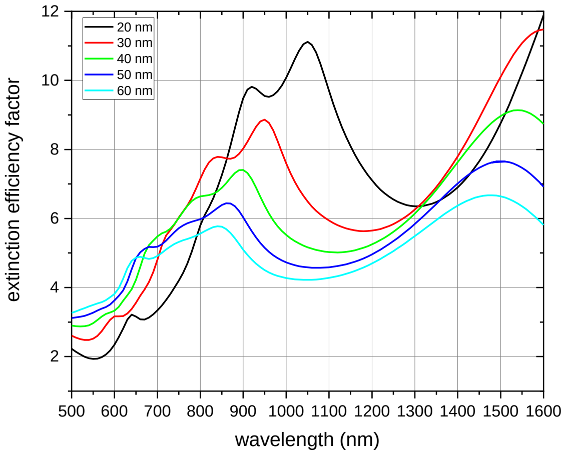

Fig. S12: Simulation results of the optical extinction efficiency factor for a nanoparticle with ellipsoidal geometry and varying short axis length in dependence of the wavelength. The main ellipsoidal axes lengths correspond to the nanoparticle with elliptical platelet geometry. The short ellipsoid length is varied according to the thicknesses of the platelet nanoparticle with a length of 20 nm shown in black, 30 nm in red, 40 nm in green, 50 nm in blue, and 60 nm in cyan. Nanoparticles are oriented randomly, and the excitation light is unpolarized.

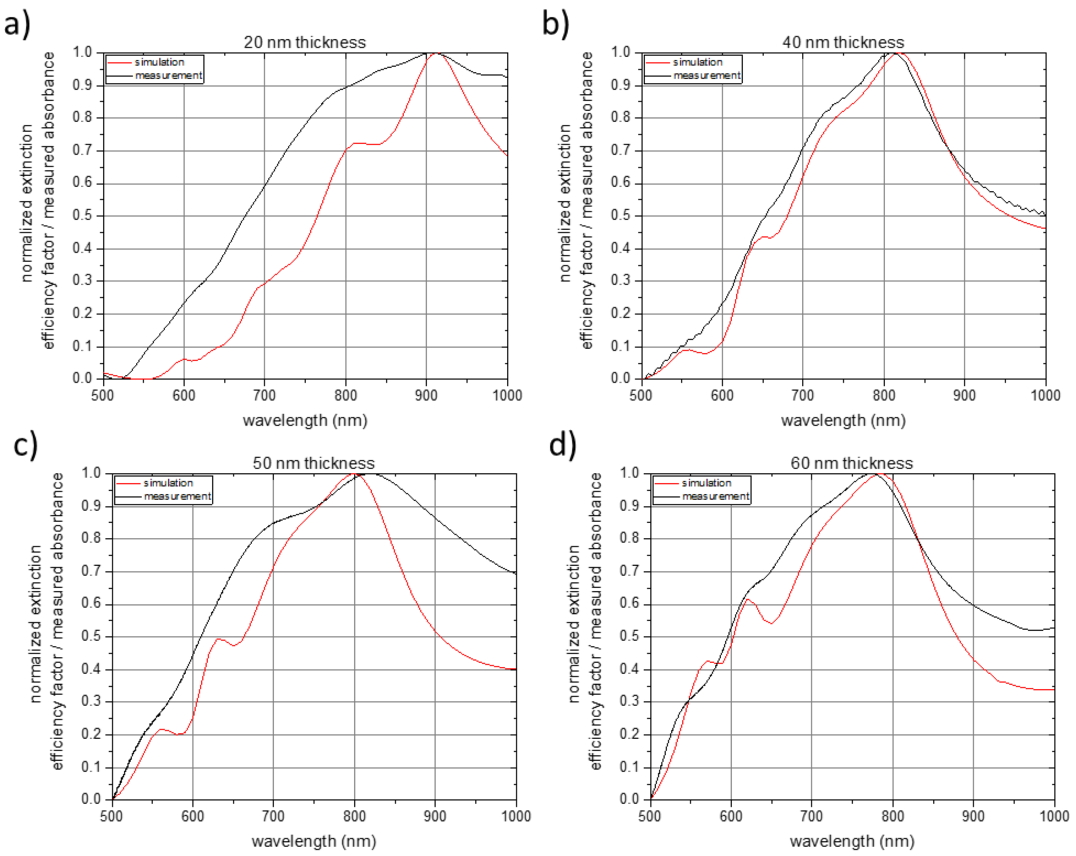

Fig. S13: Comparison of the simulated (red line) and measured (black line) absorbance spectrum for a nanoparticle of varying thicknesses. a) 20 nm thickness. b) 40 nm thickness. c) 50 nm thickness. d) 60 nm thickness. All spectra are normalized. Nanoparticles are oriented randomly, and the excitation light is unpolarized for both, the simulation and the measurement.

Table S1: Number of PEG chains per nanoparticle (NP) adopted during the synthesis (PEG/NP), total surface area of a single particle (Surface area), number of PEG molecules density per nm^2 (Theoretical PEG/nm^2), calculated number of maximum adsorbed PEG molecule per each particle (PEG$_{max}$/NP) and added amount of PEG chains per NP (PEG/NP)*.*

Sample	Radius 1 (nm)	Radius 2 (nm)	h (nm)	Surface area (nm^2)	Theoretical PEG/nm^2	PEG$_{max}$/NP	PEG/NP
NP	186.0	100.5	30.0	145 058	1.5	217 587	5 000 000

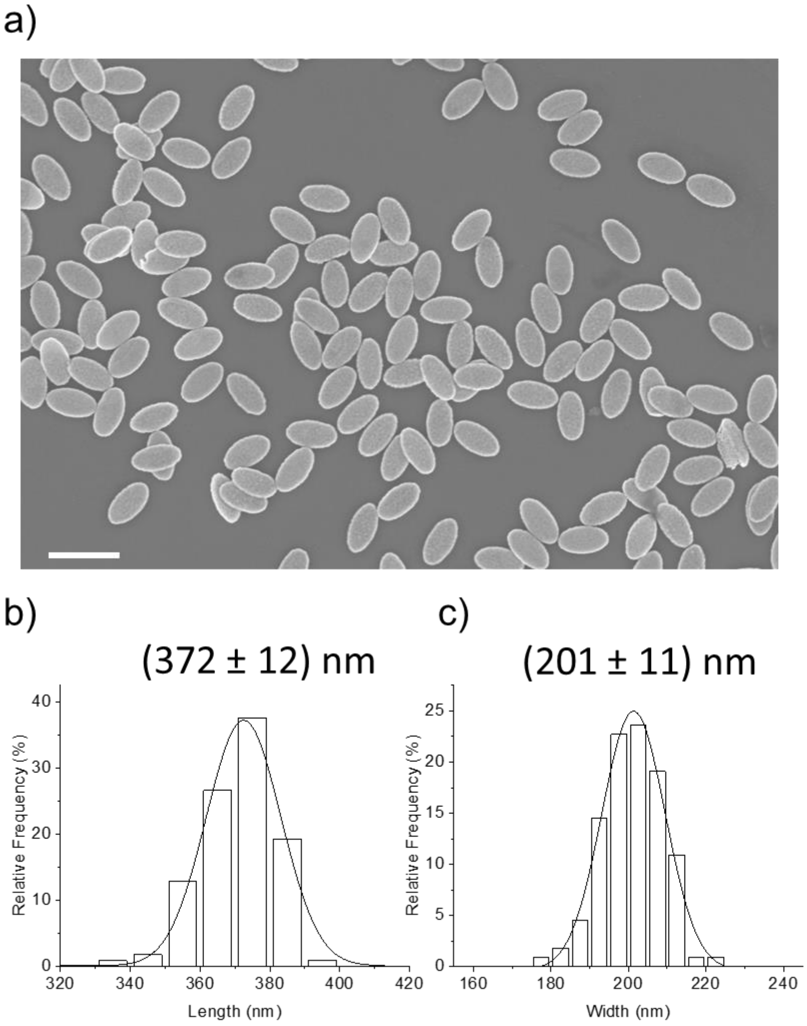

Fig. S14: a) Representative SEM micrograph of NP-COOH particles (scale bar: 500 nm), together with the relative size distribution of b) length (or Radius a) and c) width (or Radius b).

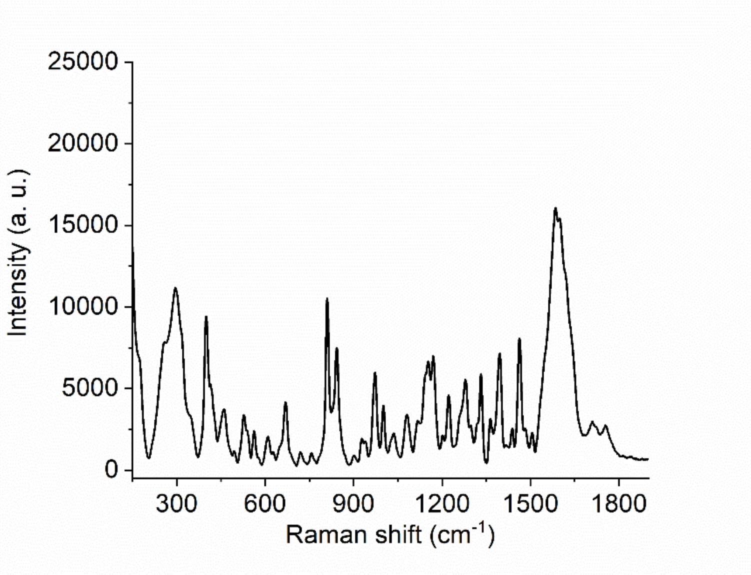

Fig. S15: Raman spectrum of PEGylated NPs.

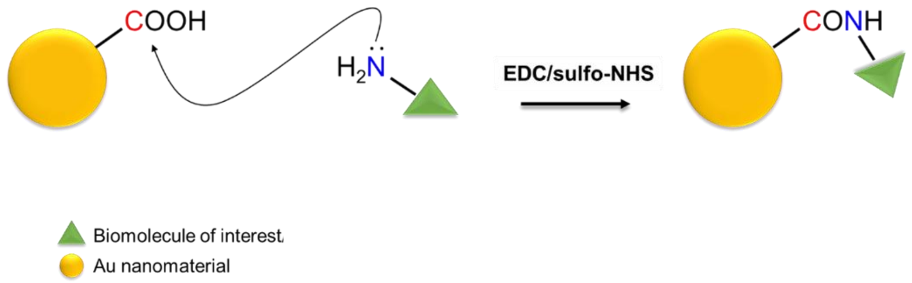

Fig. S16: Schematic representation of the reaction between the carboxylic group on gold nanomaterial and amine group on the molecule of interest (TAMRA).

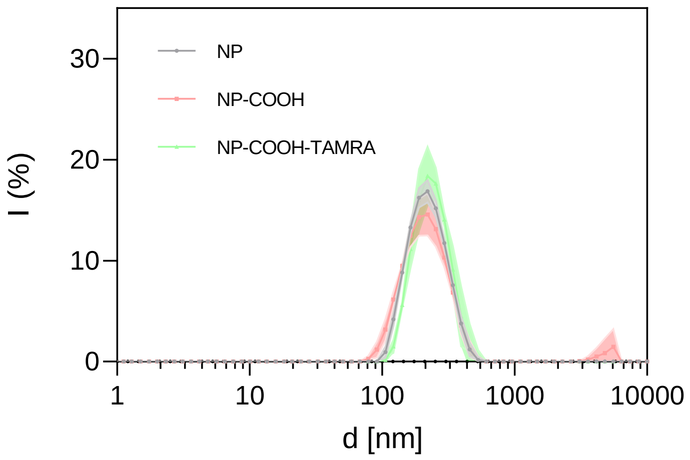

Fig. S17: Hydrodynamic diameters (by intensity) of bare NPs (black), NP-COOH (red) and NP-COOH-TAMRA (green) samples. The standard deviation is visually depicted using a shaded area proportional to the variability in the data.

Table S2: Hydrodynamic diameters (intensity and number, $D_{h,I}$ and $D_{h,N}$ respectively), polydispersity indexes (PdI) and zeta potential values (ZP) of bare NPs, NP-COOH and NP-COOH-TAMRA samples.

Sample	$D_{h,I}$ (nm)	$D_{h,N}$ (nm)	PdI	ZP (mV)
Bare NP	229.1 ± 2.8	200.4 ± 1.6	0.095 ± 0.021	- 17.2 ± 0.2
NP-COOH	220.7 ± 1.5	178.4 ± 10.9	0.333 ± 0.039	-18.1 ± 0.8
NP-COOH-TAMRA	243.6 ± 16.8	218.3 ± 7.7	0.084 ± 0.051	- 18.5 ± 0.8

Table S3: Calculation of the maximum dye molecules per NP, considering a labelling of 50% of the overall PEG.

Sample	PEG_{max}/NP	%Dye labelling	Theoretical Dye/NP
NP-COOH	217 587	50	108 794

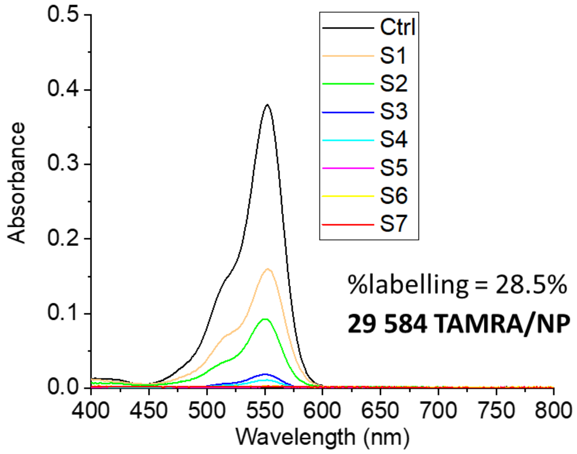

Fig. S18: Absorption spectra of TAMRA starting solution (Ctrl) and all the supernatants collected during the purification procedure of NP-COOH-TAMRA. The percentage of labelling and, thus, the amount of TAMRA per nanoparticle is evaluated by the difference between the Ctrl spectrum and the sum of the supernatants' absorption.

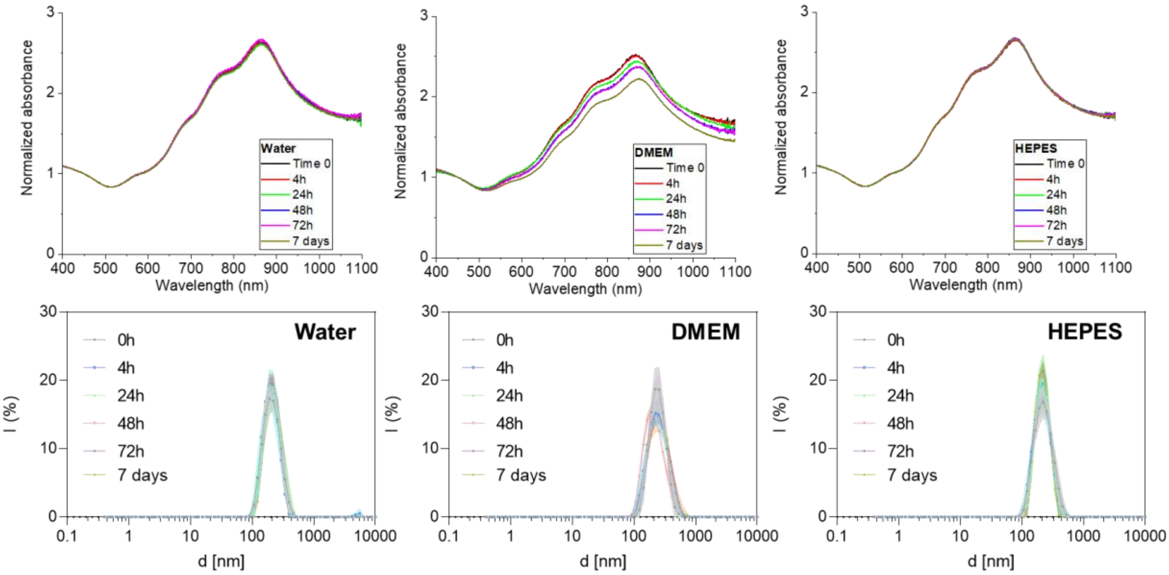

Fig. S19: Time-dependent UV-Vis spectra and DLS analysis of NP-COOH-TAMRA suspended in water, DMEM and HEPES (pH 7.4) up to 7 days.

Table S4: Hydrodynamic diameters (intensity and number) and polydispersity indexes (PdI) of NP-COOH-TAMRA sample suspended in different media (H_2O, DMEM and HEPES buffer) over time.

**System**	**Time**	**$D_{h,I}$ (nm)**	***SD***	**$D_{h,N}$ (nm)**	***SD***	**PdI**	***SD***
H_2O	0h	214.7	*7.9*	187.6	*0.7*	0.076	*0.038*
	4h	204.3	*7.4*	184.8	*12.2*	0.101	*0.104*
	24h	224.8	*11.5*	199.4	*7.3*	0.114	*0.048*
	48h	215.2	*4.2*	197.2	*2.5*	0.114	*0.027*
	72h	223.4	*9.9*	202.4	*6.5*	0.105	*0.073*
	7 days	222.7	*6.9*	203.5	*5.2*	0.117	*0.011*
DMEM	0h	249.3	*6.9*	224.5	*4.7*	0.105	*0.050*
	4h	253.4	*1.5*	215.8	*11.8*	0.126	*0.040*
	24h	250.0	*1.8*	227.7	*1.8*	0.171	*0.028*
	48h	269.5	*14.1*	224.0	*8.8*	0.149	*0.004*
	72h	261.3	*7.9*	229.7	*5.0*	0.137	*0.028*
	7 days	271.4	*5.5*	228.1	*13.4*	0.149	*0.032*
HEPES (pH 7.4)	0h	233.1	*9.6*	202.3	*3.7*	0.115	*0.017*
	4h	220.7	*5.2*	193.1	*8.3*	0.089	*0.029*
	24h	227.8	*6.5*	206.5	*4.5*	0.096	*0.041*
	48h	232.5	*8.5*	201.8	*7.8*	0.099	*0.048*
	72h	226.4	*6.3*	204.8	*10.1*	0.088	*0.010*
	7 days	216.9	*8.4*	201.2	*9.6*	0.090	*0.070*

Dimensional parameters of a single element	
Radius a (nm)	186
Radius b (nm)	100.5
Height (nm)	30
Area (cm^2)	1.45×10^{-9}
Volume (cm^3)	1.76×10^{-15}

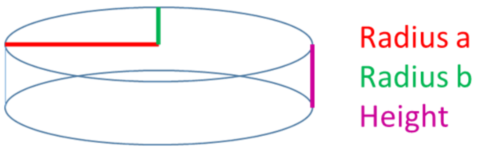

ICP results and calculation	
Line I (Au/ppm)	2.35
Line II (Au/ppm)	2.35
Dilution factor	250
$g \cdot L^{-1}$ (Au)	0.5875
Density (Au/$g \cdot cm^{-3}$)	19.32
Mass single particle (g)	3.40×10^{-14}
MW single particle ($g \cdot mol^{-1}$)	2.05×10^{10}
Concentration (nM)	0.0287

Fig. S20: Representative analysis for the extrapolation of nanoparticle concentration from ICP-OES results.

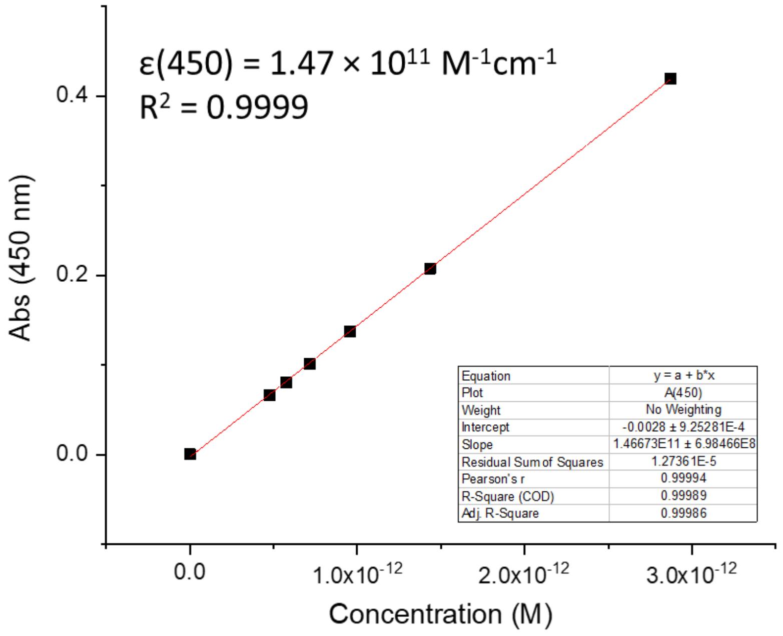

Fig. S21: Molar extinction coefficient of nanoparticles evaluated at 450 nm. The linear fit of the data is depicted with a red line.

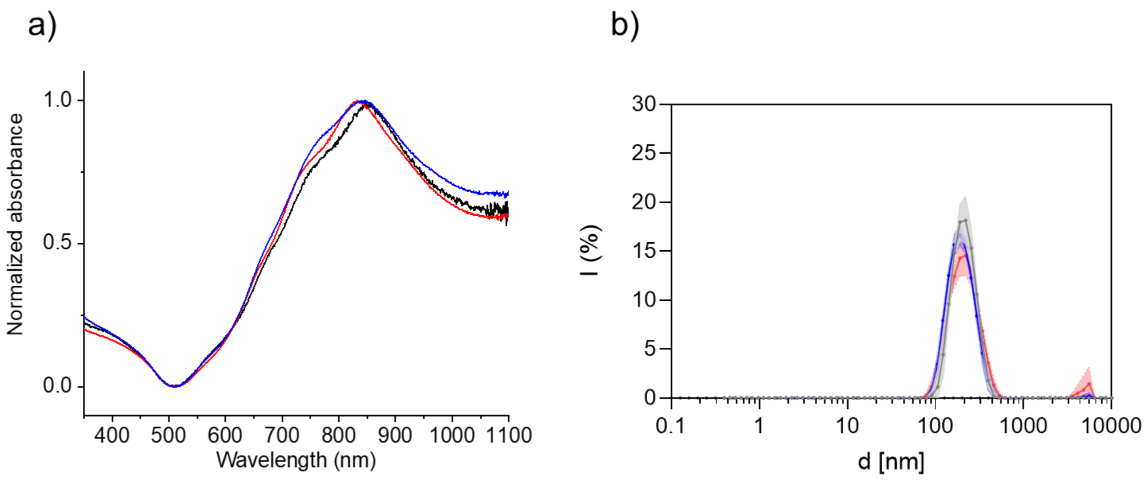

Fig. S22: a) UV-Vis spectra and b) DLS analysis of three different batches of PEGylated nanoparticles to demonstrate the high reproducibility of the coating procedure (batch#1 black line, batch#2 red line, batch#3 blue line).

Table S5: Hydrodynamic diameters (intensity and number), polydispersity indexes and zeta potential values (ZP) of different batches of PEGylated nanoparticles.

Batch	$D_{h,I}$ (nm)	$D_{h,N}$ (nm)	PdI	ZP (mV)
#1	220.7 ± 1.5	178.4 ± 10.9	0.333 ± 0.039	-18.1 ± 0.8
#2	216.8 ± 3.9	192.6 ± 12.0	0.165 ± 0.014	-20.9 ± 0.7
#3	219.4 ± 11.4	196.9 ± 4.6	0.326 ± 0.041	-17.4 ± 1.2

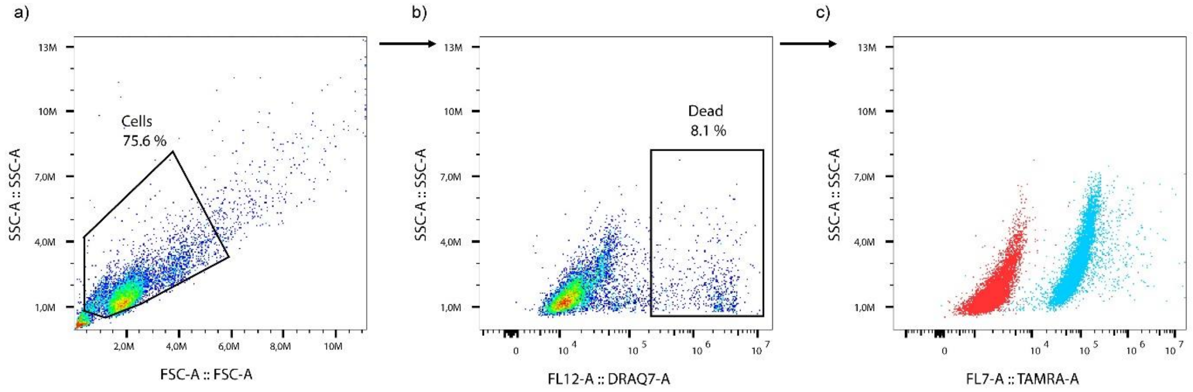

Fig. S23: Representative gating strategy and flow plots for viability determination and NPs uptake. a) Side scatter area vs. Forward scatter area plot for selecting the main cell population (75.6 % of the total events). b) Side scatter area vs. fluorescence intensity of DRAQ7™ plot (exc.: 638 nm/em.: 690(50) nm) for excluding the dead cell population (8.1 % of the total events). c) Side scatter area vs. fluorescence intensity of TAMRA plot (exc.: 561 nm/em.: 610(20) nm). In red: untreated cells. In blue: NP-COOH-TAMRA treated cells.

Table S6: Flow cytometry determination of the cellular uptake after the incubation with different concentrations of NP-COOH-TAMRA for 24 h. Data reported as median fluorescence intensity values of TAMRA (median ± s.d., n=3).

ppm	pM	MFI (TAMRA)
0	0	1973.67 ± 47.61
1	0.05	7573.67 ± 568.60
3	0.15	17953.33 ± 1389.04
10	0.49	46791.67 ± 886.63
30	1.46	90519.33 ± 3903.78
100	4.88	185132.67 ± 4769.04
300	14.63	449333.33 ± 6806.86
